# A Passive Decomposition Based Robust Synchronous Motion Control of Multi-Motors and Experimental Verification

**DOI:** 10.3390/s23177603

**Published:** 2023-09-01

**Authors:** DaeYi Jung, Seulgi Kim

**Affiliations:** 1 School of Mechanical and Automotive Engineering, Kunsan National University, Gunsan-si 54150, Republic of Korea; 2 KOCETI, 36 Sandan-ro, Gunsan-si 54004, Republic of Korea; sgkim@kunsan.ac.kr

**Keywords:** synchronous motion control, passive decomposition, locked and shape systems, high-order sliding mode control, multiple motors, parametric uncertainty, external disturbance

## Abstract

Recently, with the trend of redundancy design, the importance of synchronous motion control of multiple motors has been emphasized in various fields such as automotive, construction, and industrial engineering. Therefore, this paper proposed a novel passive decomposition-based robust synchronous control strategy for a multi-motor system, guaranteeing that both the tracking error of each motor and the synchronous error between motors are ultimately and synchronously bounded, even under the presence of parametric uncertainty and unstructured external disturbance. Specifically, a passive decomposition is used to obtain the locked and shape systems from the original system, and then a sliding mode control system along with robust compensations is designed for each decomposed system to achieve the precise synchronous motion control of the *n* number of motors. Here, the controller for the locked system reduces the tracking errors of motors for a given desired trajectory, while the controller for the shaped system decreases the synchronous errors between motors. Furthermore, the control system is generally and conveniently formulated to adopt the arbitrary *n* number of motors that must track a given desired trajectory and be synchronized. Compared to other related studies, this work especially focused on increasing the robustness of the entire system using both high-order sliding mode control and two separate compensation terms for model uncertainty and unstructured external disturbance. Finally, to validate the effectiveness of the proposed synchronous control strategy, the extensive experimental studies on two/three/four-geared BLDC motors with a high dead-zone effect were conducted, and we also compared the synchronous control performance of the proposed control strategy with the other representative control approaches, a master–slave control scheme and an independent one to address the superiority of the proposed control system. Regardless of the number of motors, due to the robustness of the control system, it is found that the proposed control ensures the tracking and synchronous errors are less than 1 degree for the sine-wave trajectory while it guarantees that the errors are below 1.5 degree for the trapezoidal trajectory. This control approach can be widely and generally applied to the multiple motor control required in various engineering fields.

## 1. Introduction

To generate one perfect motion using the multiple motors (or actuators), the synchronous motion control has long been an important research topic, and its importance has been emphasized with the recent trend of redundancy design in many mechanical-electrical systems such as automotive, construction, and industrial active systems. For instance, the recent dual-motor-based Steer-by-Wire system in the vehicle requires the synchronous angular position control between two motors to guarantee the reliable and robust fault-tolerant control and produce a large torque [1,2,3,4,5]. Ref. [1] employed the coordinated control of dual steering motors. With dual motor-microcontroller architecture, ref. [2] developed a control algorithm, allowing the system to reconfigure itself automatically in the event of a single point fault without degrading the control system performance. Ref. [3] proposed dual-servo synchronization motion control for the angular position tracking of the road wheel reference input by controlling two actuators synchronously and cooperatively.

Refs. [1,2,3] provides the fundamental structure for the synchronous motion control of a multi-motor but have no treatment of model uncertainty and external disturbance.

Furthermore, ref. [4] proposed the novel master–slave control scheme using both a continuous sliding mode control and a disturbance observer to ensure strong robustness against model uncertainties and external disturbances, but it requires an additional effort for designing a disturbance observer. Ref. [5] guaranteed a precise, stable, and fast response for the collaborative control of multiple motors using both the conventional PID controller and a radial basis function neural (RBF) network for tuning processing of the PID controller. Here, the control performance increased due to flexible gain of PID via RBF but it will be difficult to implement this scheme with a cost-effective microcontroller.

The synchronous control of a multi-motor is also applied to the conveyer belt system and continuous production line system [6,7]. Ref. [6] presented the practical control strategy of multi-motor drives of high-power belt conveyors and [7] designed the fuzzy model-based optimal control of a continuous production line using multi-motors.

Furthermore, this synchronous motion control technique is used for the driving system in an electrical vehicle [8,9] and a robotic manipulator [10], as well as a gantry crane system [11]. Ref. [8] points out that the electrical vehicle must be a fault-tolerant system, (i.e., multi-motor-based driving system) and [9] described the implementation of the electrical vehicle drive control algorithm with torque distribution on an FPGA platform. Ref. [10] proposed the cross-coupling ring control based on the fuzzy theory (CRCF) for the multi-motor coordinated control of intelligent robots. Ref. [11] showed the application of adjustable speed induction motor drives for the gantry cranes. Regarding [6,7,8,9,10,11], due to the absence of (or partial) compensation for parametric uncertainty and external disturbance, the control performance will be sensitive to those disturbing effects.

Moreover, for a decade, many synchronous control schemes of multi-motor systems have been proposed [12,13,14,15,16,17,18,19,20,21]. Refs. [12,13,14] proposed the passivity based synchronous control approach for a tele-operated manipulator system by introducing the concept of passivity and the passive decomposition technique. Ref. [12] guarantees synchronized motion between master and slave using the passivity observer and controller. Ref. [13] investigated a passive bilateral feed-forward control scheme for linear dynamically similar (LDS) tele-operated master–slave manipulator. The proposed technique is robust for model uncertainty and inaccuracy of force measurements and individually secures the aspects for the coordination error and overall motion. Furthermore, ref. [14] proposed a passive bilateral tele-operation synchronous control law for the multiple DOF nonlinear master–slave robotic systems using the passive decomposition for 2n-DOF nonlinear tele-operated dynamics without contravening passivity. Although [12,13,14] are pioneering works for synchronous motion control for tele-operating manipulator systems using the concept of passive decomposition, the effectiveness and robustness of designed controllers have been tested with advanced and expensive motors (vs. cost-effective ones featured with a high dead-zone property).

In line with [12,13,14,15,16], they applied the cross-coupling scheme structure to the synchronous speed control of multiple-motor. Ref. [15] presents a control scheme of synchronous motion based on the artificial potential field and cross-coupled structure and, ref. [16] introduced an adjacent cross-coupling synchronous control to address the problem of phase and speed synchronization control of multi-exciters in vibration. Even though well-structured control systems using every synchronous combination between agents are developed in [15,16], the effectiveness of the control systems was only validated through a semi-physical model [15] or simulation [16].

Refs. [17,18,19,20,21,22,23,24,25,26,27] used the sliding mode control technique, fuzzy sliding mode control, and adaptive control to achieve the synchronous motion control. Ref. [17] described the fuzzy adaptive sliding mode controller for uncertain nonlinear multi-motor systems to address the chattering problem in the two-motor synchronization problem. Ref. [18] designed a sliding mode (SM) feedback linearization control system for a multi-motors web winding system. Refs. [17,18] presented only simulation results based on limited control scenarios and the direct extension of a control strategy for *n* arbitrary agents is also doubtful. Ref. [19] proposed the multi-motor improved relative coupling cooperative control based on a sliding mode controller, and showed a more significant control effect on the system error of each motor in comparison with the traditional relative coupling control structure. However, the effectiveness of the control system was only validated through the tracking of constant speed. Ref. [20] used the second Lyapunov method together with a reference model to ensure asymptotic stability of a Continuous Strip Processing Line with Multi-Motor Drive but requires relatively exact parameters of the system to achieve accurate control. Ref. [21] proposes an adaptive output feedback controller for the multi-motor driving system to guarantee all the tracking errors constrained within the prescribed bounds. And, a modified barrier Lyapunov function (MBLF) is applied to derive the adaptive law in the proposed control system. Here, the adaptive tuning law could lead to unwanted results if the system runs with the control scenario containing no (or a little) persistent excitation. Furthermore, ref. [22] presented a hybrid adaptive fuzzy multi-agent consensus scheme for leader-follower multi-motor speed coordination. Here, it is found that the performance of the control system highly depends on the fuzzy sets, thus the sets may be tuned whenever the desired trajectory is changed. Ref. [23] proposed an adaptive robust H-infinity control scheme, combining a robust tracking controller with a distributed synchronization controller, to guarantee both the load tracking and synchronization. Ref. [24] designed an adaptive control strategy based on the optimal sliding surface for multi-motor driving systems along with a leaky echo state network-based observer. Due to the complicated design of the control system and observer in [23,24], the practical application of [23,24] may be challenging as a cost-effective micro-controller. Recently, ref. [25] proposed synchronous motion control between the actuators in a 2-DOF tele-operating system using passive decomposition, sliding mode control as well as an RLS filter to deal with the dead-zone effect of the actuator. Ref. [25] requires extra effort to estimate the dead-zone parameters via the RLS filter.

To maximize the control performance of a PMSM, a new IDA-PBC paired with a high order sliding mode and non-linear observer technique is proposed in [26]. In [27], a novel generalized non-linear robust predictive controller has been explored for aiming the tracking reference speed of PMSM, ensuring robustness to external disturbances and parameters. However, the validity of proposed schemes in [26,27] must be tested with an actual implementation containing various control scenarios since [26] used relatively high control gains and [27] is designed under the assumption that the disturbance is slow and has a simple scenario. Ref. [28] proposed a novel control strategy using a composite sliding mode observer (back-EMF error extraction) with a modified feed-forward phase-locked loop (PLL) for ensuring high accuracy position and speed control of shaftless RDT (rim-driven thruster) motors. The limitation of [28] relies on the fact that the rate of change of the motor speed is small when designing the observer, and the validity of the proposed method is investigated only by simulation.

Ref. [29] provides the holistic reviews of the multi-motor control strategies for automotive applications along with the fault-tolerant multi-motor drive topologies.

In this paper, in the line with many control techniques [12,13,14,15,16,17,18,19,20,21,22,23,24,25,26,27,28], based on the ideas of both the passive decomposition [12,13,14] and sliding mode control techniques [17,18,19,20,21,25] without any adaptive compensation law [4,5,17,21,22,23,24], a novel passive decomposition-based robust synchronous motion control of multi-motors is presented.

Specifically, based on a passive decomposition, the locked and shape system is achieved from the original system. This implies that the controllers for each system (locked + shaped system) reduce not only the tracking errors (locked motion) of motors for a given desired trajectory but also the synchronous errors between motors (shape motion).

And then a robust high-order sliding mode control along with additional compensation components for model uncertainty and external disturbance is designed for each decomposed system. Unlike [17,18,19], the high-order sliding surface (including an integral of error) used here attenuates the influence of external disturbances more effectively than a control using a standard first-order sliding surface. This point of view is briefly mentioned in Appendix A.

In this study, besides the high-order sliding mode control, the control system additionally contains two separate compensation terms for rejecting both model uncertainty and external disturbance to achieve further robustness of the entire system.

These two compensation terms are designed by a simple but effective signum (or sat) function. Therefore, the control system proposed here is designed in a relatively pragmatic manner for better implementation in cost-effective ECUs by avoiding the inclusion of any adaptive control strategies (i.e., the integral type of adaptive tuning law) consuming more computational load and sometimes leading in the wrong direction when the tuning law produces unwanted outcomes.

The final control law is obtained via a transformation matrix from locked and shape coordinates to the original one. Furthermore, the technique presented here is systematically formulated to use and adopt the arbitrarily *n* number of agents that must be synchronously controlled and track the desired trajectory. The frame of this technique can be generally extended to any multi-motor driving setting.

Compared to several works [15,16,17,18,22,23,26,27], to validate the effectiveness of the proposed synchronous control strategy, the extensive experimental studies on 2/3/4-geared cost-effective BLDC motors were also performed based on two representative control scenarios (sine-wave and trapezoidal trajectories).

Moreover, the other representative control approaches, a master–slave control scheme and an independent one, were introduced here, and the proposed control method was compared and evaluated with these control methods.

According to the contributions above, this work will be a valuable asset for those who wish to systematically design the synchronous motion control of a multi-motor system in any field.

The rest of this paper is as follows. Section 2 and Section 3 present the problem formulation and passive decomposition technique, respectively. Furthermore, the synchronous control scheme is described in Section 4, other control approaches are presented in Section 5, and the experimental tests and results are included in Section 6. Finally, the conclusions are remarked.

## 2. Problem Formulation

This section introduces the mathematical model for *n* motors to be synchronously controlled. Consider E.O.M of *n* number of motors,

(1a) $$J_{m.i}{\ddot{\theta }}_{i}\left(t\right)+B_{m.i}{\dot{\theta }}_{i}\left(t\right)=\tau_{i}\left(t\right)+d_{i}\left(t\right)\;\mathrm{for}\;i=1,2,3,\cdots ,n$$

(1b) $$\left\{\begin{array}{c} J_{m.1}{\ddot{\theta }}_{1}\left(t\right)+B_{m.1}{\dot{\theta }}_{1}\left(t\right)=\tau_{1}\left(t\right)+d_{1}\left(t\right)\\ J_{m.2}{\ddot{\theta }}_{2}\left(t\right)+B_{m.2}{\dot{\theta }}_{2}\left(t\right)=\tau_{2}\left(t\right)+d_{2}\left(t\right)\\ \begin{array}{c} \vdots \\ J_{m.n}{\ddot{\theta }}_{n}\left(t\right)+B_{m.n}{\dot{\theta }}_{n}\left(t\right)=\tau_{n}\left(t\right)+d_{n}(t) \end{array} \end{array}\right.$$

where $\theta_{i}$ (rad) (for *i =* 1, 2, … *n*) is the rotational angular positions of motors. And $J_{m.i}$ ($kg\;m^{2}$) and $B_{m.i}$ ($kg\;m^{2}/s$) (for *i =* 1, 2, … *n*) are the inertia and the viscous damping coefficient of motors, respectively. Here, even though $J_{m.i}$ can be fairly determined via the design aids such as CAD/INVENTOR, the parametric uncertainty of $B_{m.i}$ cannot be negligible, so this study will be concerned with this aspect in designing the control system. Thus, it is assumed that $\left|B_{m.i}-{\hat{B}}_{m.i}\right|\leq k\in R$, where ${\hat{B}}_{m.i}$ is the nominal value of $B_{m.i}$.

Also, $\tau_{i}\left(t\right)$ (*Nm*) and $d_{i}\left(t\right)$ (*Nm*) (for *i =* 1, 2, … *n*) represent the control torques and the unstructured external disturbances, respectively. It is also assumed that $d_{i}\left(t\right)$ is bounded such that $\left|d_{i}\left(t\right)\right|\leq d_{o}\in R$.

The set of equations in (1) can be formulated as,

(2) $$M\ddot{X}\left(t\right)+C\dot{X}\left(t\right)=T\left(t\right)+D\left(t\right)$$

where $X={\left[\begin{array}{ccc} \begin{array}{cc} \theta_{1} & \theta_{2} \end{array} & \cdots & \theta_{n} \end{array}\right]}^{T}\in R^{n\times 1}$ is the rotational angular position vector of the motors. Also, $M>0=diag\left[\begin{array}{ccc} \begin{array}{cc} J_{m.1} & J_{m.2} \end{array} & \cdots & J_{m.n} \end{array}\right]\in R^{n\times n}$ and $C>0=diag\left[\begin{array}{ccc} \begin{array}{cc} B_{m.1} & B_{m.2} \end{array} & \cdots & B_{m.n} \end{array}\right]\in R^{n\times n}$ are the inertia matrix and the viscous damping matrix, respectively. In addition, $T\left(t\right)=\left[\begin{array}{ccc} \begin{array}{cc} \tau_{1} & \tau_{2} \end{array} & \cdots & \tau_{n} \end{array}\right]\in R^{n\times 1}$ and $D\left(t\right)=\left[\begin{array}{ccc} \begin{array}{cc} d_{1} & d_{2} \end{array} & \cdots & d_{n} \end{array}\right]\in R^{n\times 1}$ are the vectors for the control torques of motors and the external disturbances, respectively. It is also clear that $\left\|D\left(t\right)\right\|\leq D_{o}\in R$ due to $\left|d_{i}\left(t\right)\right|\leq d_{o}\in R$.

**Remark** **1.** *This study proposed a synchronous control system to achieve perfect motion synchronization between the motors and tracking the desired trajectory (i.e., $\begin{array}{ccc} \begin{array}{cc} \theta_{1} & =\theta_{2} \end{array}= & \cdots & =\theta_{n} \end{array}=\theta_{d}$, where $\theta_{d}$ is a given desired trajectory). If each motor has a different gear ratio, the synchronous solution can be $\rho_{1}\begin{array}{ccc} \begin{array}{cc} \theta_{1} & =\rho_{2}\theta_{2} \end{array} & \cdots & =\rho_{n}\theta_{n}=\rho_{d}\theta_{d} \end{array}$, (where $\rho_{i}\in R$ for i = 1,2, … n ) are the constant scaling factors. However, it is assumed that each motor has an identical gear ratio for this study.*

## 3. Passive Decomposition

In this section, we decomposed the set of dynamics in (2) into two systems according to two aspects, gross motion (i.e., locked system) and coordination (i.e, shape system). Ref. [12] shows that, for LDS, the two decomposed systems can be individually controlled, and as long as the individual controller guarantees that each closed-loop system is energetically passive, the combined system is also energetically passive.

There is only one gross motion (i.e., a locked coordinate) regardless of how many agents are synchronously controlled. However, for *n* agents, the number of possible coordinates in the shape system can have multiple choices. In this study, we consider all combinations between the two agents (i.e., fully cross-coupled network structure).

**Figure 1:**
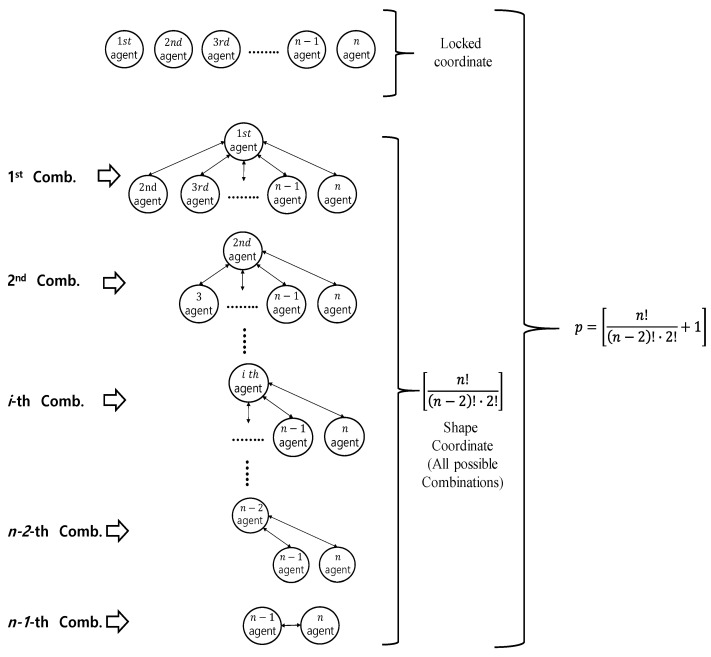
A locked coordinate and every combination between two agents for shape coordinate.

Therefore, Figure 1 shows *n* agents to be controlled, describing one case for a locked system and all possible combinations of pairs between agents for the shape system.

Specifically, the entire coordinates, a locked one and the shaped ones, are given by [12,13,25]

(3) $$q\left(t\right)={\left[\begin{array}{cc} q_{L}\left(t\right) & q_{S} \end{array}\left(t\right)\right]}^{T}$$

(4) $$q_{L}\left(t\right)=1/n\sum_{i=1}^{n}\theta_{i}\left(t\right)$$

(5) $$q_{S}\left(t\right)={\left[\begin{array}{ccc} \theta_{1/2} & \theta_{1/3} & \begin{array}{ccc} \cdots & \theta_{1/n} & \begin{array}{ccc} \theta_{2/3} & \theta_{2/4} & \begin{array}{ccc} \cdots & \theta_{2/n} & \begin{array}{ccc} \theta_{3/4} & \theta_{3/5} & \begin{array}{ccc} \cdots & \theta_{3/n} & \begin{array}{ccc} \cdots \cdots & \theta_{\left(n-2\right)/(n-1)} & \begin{array}{cc} \theta_{\left(n-2\right)/n} & \theta_{\left(n-1\right)/n} \end{array} \end{array} \end{array} \end{array} \end{array} \end{array} \end{array} \end{array}\right]}^{T}$$

where $q_{L}\left(t\right)\in R$ is a locked coordinate and $q_{s}\left(t\right)\in R^{(p-1)\times 1}$ is the shaped coordinate vector.

And, the notation $\theta_{m/n}$ in (5) is the difference between angular rotational positions of motors, $\theta_{m}-\theta_{n}$, the *m*-th and *n*-th ones in every combination.

Based on (4) and (5), proposing the transformation matrix from the physical coordinate $X\left(t\right)$ to the locked-shape coordinates,

(6) $$H=\left[\begin{array}{c} \begin{array}{c} \begin{array}{c} \begin{array}{c} {℧}_{1\times n}\\ \begin{array}{cc} A_{n-1\times 1} & -I_{n-1\times n-1} \end{array} \end{array}\\ \begin{array}{ccc} O_{n-2\times 1} & A_{n-2\times 1} & -I_{n-2\times n-2} \end{array} \end{array}\\ \begin{array}{ccc} O_{n-3\times 2} & A_{n-3\times 1} & -I_{n-3\times n-3} \end{array} \end{array}\\ \vdots \\ \begin{array}{ccc} O_{2\times n-3} & A_{2\times 1} & -I_{2\times 2} \end{array}\\ \begin{array}{ccc} O_{1\times n-2} & 1 & -1 \end{array} \end{array}\right]\in R^{p\times n}$$

where the sub-notation *p* in (6) is defined as $p=\left[\frac{n!}{\left(n-2\right)!\cdot 2!}+1\right]$.

And, ${℧}_{1\times n}=1/n\left[\begin{array}{ccc} \begin{array}{cc} 1 & 1 \end{array} & \cdots & 1 \end{array}\right]$ and $A_{i\times 1}={\left[\begin{array}{ccc} \begin{array}{cc} 1 & 1 \end{array} & \cdots & 1 \end{array}\right]}^{T}$ for *i* = 2,⋯n−1. Both O and I are zero matrices and identity matrices with the proper dimensions specified in (6).

Using (6), the relation between $q\left(t\right)$ and the physical coordinates, $X\left(t\right)$, is given by

(7) $$q\left(t\right)={\left[\begin{array}{cc} q_{L}\left(t\right) & q_{S} \end{array}\left(t\right)\right]}^{T}=HX\left(t\right)\in R^{p\times 1}$$

Furthermore, (7) becomes

(8) $$X\left(t\right)=\Gamma q\left(t\right)\in R^{n\times 1}$$

where ${{\Gamma =(H^{T}H)}^{-1}H}^{T}\in R^{n\times p}$. And it should be noted that the pseudo inverse matrix ${\left(H^{T}H\right)}^{-1}\in R^{n\times n}$ (i.e, rank($H^{T}H$) = *n*) is used due to the fact that H may not be a square matrix (i.e, p≠n). If H is a square matrix, the inverse matrix will be used.

Using (8), the original dynamics can be transformed into the locked and shape systems.

On the other hand, another form of (2) is given by

(9) $$\ddot{X}\left(t\right)=-M^{-1}C\dot{X}\left(t\right)+M^{-1}T\left(t\right)+M^{-1}D\left(t\right)$$

Substituting (8) into (9) and then multiplying $\Gamma^{T}$ to the both sides of the result yield

(10) $$\Gamma^{T}\Gamma \ddot{q}\left(t\right)=-\overset{̿}{C}\dot{q}\left(t\right)+\overset{̿}{T}\left(t\right)+\overset{̿}{D}\left(t\right)$$

where $\overset{̿}{C}=\Gamma^{T}M^{-1}C\Gamma \in R^{p\times p}$, $\overset{̿}{T}(t)=\Gamma^{T}M^{-1}T(t)\in R^{p\times 1}$, and

$$\overset{̿}{D}(t)=\Gamma^{T}M^{-1}D(t)\in R^{p\times 1}.$$

And, the partitions of matrices and vectors according to $q_{L}\left(t\right)$ and $q_{S}(t)$ in (10) is shown,

(11) $$\Gamma^{T}\Gamma =\left[\begin{array}{cc} n & O_{1\times (p-1)}\\ O_{(p-1)\times 1} & \Upsilon_{\left(p-1)\times (p-1\right)} \end{array}\right]\in R^{p\times p}\mathrm{and}\;\;\overset{̿}{C}=\left[\begin{array}{c} \begin{array}{cc} {\overset{̿}{C}}_{L} & {\overset{̿}{C}}_{LS} \end{array}\\ \begin{array}{cc} {\overset{̿}{C}}_{SL} & {\overset{̿}{C}}_{S} \end{array} \end{array}\right]\in R^{p\times p}$$

(12) $$\overset{̿}{D}={\left[\begin{array}{cc} {\overset{̿}{D}}_{L}\left(t\right) & {\overset{̿}{D}}_{S}\left(t\right) \end{array}\right]}^{T}\mathrm{and}\;\;\overset{̿}{T}\left(t\right)={\left[\begin{array}{cc} {\overset{̿}{T}}_{L}\left(t\right) & {\overset{̿}{T}}_{S}\left(t\right) \end{array}\right]}^{T}$$

For clear understanding, the dimension of each component in (11) and (12) is specified: ${\overset{̿}{C}}_{L}\in R$**,** ${\overset{̿}{C}}_{LS}\in R^{1\times (p-1)}$**,** ${\overset{̿}{C}}_{SL}\in R^{(p-1)\times 1}$**,** ${\overset{̿}{C}}_{S}\in R^{\left(p-1\right)\times (p-1)}$**,**${\overset{̿}{D}}_{L}\left(t\right)\in R$, ${\overset{̿}{D}}_{S}\left(t\right)\in R^{(p-1)\times 1}$, ${\overset{̿}{T}}_{L}\left(t\right)\in R$, and ${\overset{̿}{T}}_{S}\left(t\right)\in R^{(p-1)\times 1}$.

Specifically, based on (11) and (12), (10) becomes

(13) $${n\ddot{q}}_{L}\left(t\right)=-{\overset{̿}{C}}_{L}{\dot{q}}_{L}\left(t\right)-{\overset{̿}{C}}_{LS}{\dot{q}}_{s}\left(t\right)+{\overset{̿}{T}}_{L}\left(t\right)+{\overset{̿}{D}}_{L}\left(t\right)$$

(14) $${\Upsilon \ddot{q}}_{S}\left(t\right)=-{\overset{̿}{C}}_{S}{\dot{q}}_{S}\left(t\right)-{\overset{̿}{C}}_{SL}{\dot{q}}_{L}\left(t\right)+{\overset{̿}{T}}_{S}\left(t\right)+{\overset{̿}{D}}_{S}\left(t\right)$$

(13) and (14) represent the decomposed system, a locked system, and a shape system, respectively. In the next Section, each control system for the decomposed system in (13) and (14) will be designed using sliding mode control and robust compensation term for rejecting both parametric uncertainty and external disturbance.

## 4. Synchronous Control System

This section proposes the synchronous control system based on the locked-shape system in (13) and (14). In the locked system, a gross motion should track the desired trajectory while the difference of motion between two agents in every combination should be minimized in the shape system. In addition, the system should be robust for the parametric uncertainty and the unstructured but bounded external disturbance. Meanwhile, the stability of system with the proposed controller should also be guaranteed (i.e, energetically passive).

### 4.1. Controller Design of Locked System

This sub-section presents the controller design of the locked system and shows the stability analysis of the proposed controller via Lyapunov approach. Here, the high-order sliding mode control and robust compensation terms are used to reject the parametric uncertainty and external disturbances.

From (13), we have

(15) $${\ddot{q}}_{L}\left(t\right)=-\frac{1}{n}[{\overset{̿}{C}}_{L}{\dot{q}}_{L}\left(t\right)+{\overset{̿}{C}}_{LS}{\dot{q}}_{s}\left(t\right)]+\frac{1}{n}[{\overset{̿}{T}}_{L}\left(t\right)+{\overset{̿}{D}}_{L}\left(t\right)]$$

Based on (15), proposing the control law such that

(16) $$\begin{array}{c} {\overset{̿}{T}}_{L}\left(t\right)=n{\ddot{q}}_{L.d}\left(t\right)+\tilde{{\overset{̿}{C}}_{L}}{\dot{q}}_{L}\left(t\right)+\tilde{{\overset{̿}{C}}_{LS}}{\dot{q}}_{s}\left(t\right)-n[3\lambda_{L}\dot{\varepsilon_{L}\;}\left(t\right)+3{\lambda_{L}}^{2}\varepsilon_{L}\left(t\right)+\\ {\lambda_{L}}^{3}\int \varepsilon_{L}\left(t\right)dt]{+\overset{̿}{U}}_{L.R}\left(t\right)+{\overset{̿}{U}}_{L}\left(t\right) \end{array}$$

(17) $${\overset{̿}{U}}_{L.R}\left(t\right)=\left\{\begin{array}{c} -\frac{k_{L}{\left|{\dot{q}}_{L}\left(t\right)\right|S}_{L}\left(t\right)}{\left|S_{L}\left(t\right)\right|}-\frac{k_{LS}\left\|{\dot{q}}_{s}\left(t\right)\right\|S_{L}\left(t\right)}{\left|S_{L}\left(t\right)\right|}\\ 0 \end{array}\right.\begin{array}{c} \;\;if\left|S_{L}\left(t\right)\right|\geq \Phi_{L}\\ \;\;if\left|S_{L}\left(t\right)\right|<\Phi_{L} \end{array}$$

(18) $${\overset{̿}{U}}_{L}\left(t\right)=\left\{\begin{array}{c} -\gamma_{L}S_{L}(t)/\left|S_{L}(t)\right|\\ -\gamma_{L}S_{L}(t)/\Phi_{L} \end{array}\right.\begin{array}{c} \;\;if\left|S_{L}(t)\right|\geq \Phi_{L}\\ \;\;if\left|S_{L}(t)\right|<\Phi_{L} \end{array}$$

(19) $$\begin{array}{c} S_{L}(t)={\dot{\varepsilon }}_{L}\left(t\right)+2\lambda_{L}\varepsilon_{L}\left(t\right)+{\lambda_{L}}^{2}\int \varepsilon_{L}\left(t\right)dt \end{array}$$

where $\varepsilon_{L}\left(t\right)=q_{L}\left(t\right)-q_{L.d}\left(t\right)\in R$ is the error between the locked coordinate and the desired trajectory, and $S_{L}(t)\in R$ in (19) is the sliding surface, which is a function of the locked error coordinate $\varepsilon_{L}\left(t\right)$. Also, $\tilde{{\overset{̿}{C}}_{L}}$ and $\tilde{{\overset{̿}{C}}_{LS}}$ are the nominal values of ${\overset{̿}{C}}_{L}$ and ${\overset{̿}{C}}_{LS}$ (which are a function of $B_{m.i}$ (parametric uncertainty)), and, due to $\left|B_{m.i}-{\hat{B}}_{m.i}\right|\leq k$, it is assumed that $\left|\tilde{{\overset{̿}{C}}_{L}}-{\overset{̿}{C}}_{L}\right|\leq k_{L}$ and $\left\|\tilde{{\overset{̿}{C}}_{LS}}-{\overset{̿}{C}}_{LS}\right\|\leq k_{LS}$, where the constant gains $k_{L}\in R$ and $k_{LS}\in R$ are the known positive upper values for each.

And, $\lambda_{L}>0\in R$ and $\gamma_{L}>0\in R$ are positive constant gains, and $\Phi_{L}>0\in R$ is the thickness of boundary layer.

Here, (17) is the compensation term for the model uncertainty and (18) counteracts for the external disturbance.

**Theorem** **1.** *As long as the parametric uncertainty and external disturbance are bounded such as* $\left|{\overset{̿}{D}}_{L}\left(t\right)\right|\leq {∆}_{L}\in R$, $\left|{\Delta \overset{̿}{C}}_{L}\right|\leq k_{L}$, *and* $\left\|\Delta {\overset{̿}{C}}_{LS}\right\|\leq k_{LS}$, *the locked system (15) is ultimately bounded by the control law in (16) through (18). where* ${\Delta \overset{̿}{C}}_{L}=\tilde{{\overset{̿}{C}}_{L}}-{\overset{̿}{C}}_{L}$ *and* $\Delta {\overset{̿}{C}}_{LS}=\tilde{{\overset{̿}{C}}_{LS}}-{\overset{̿}{C}}_{LS}$.

**Proof.**  Substituting (16) into (15), the closed-loop locked system is given by

(20) $$\begin{array}{c} {\ddot{\varepsilon }}_{L}\left(t\right)=\frac{1}{n}\left[{\Delta \overset{̿}{C}}_{L}{\dot{q}}_{L}\left(t\right)+\Delta {\overset{̿}{C}}_{LS}{\dot{q}}_{s}\left(t\right)+{\overset{̿}{U}}_{L.R}\left(t\right)\right]-\left[3\lambda_{L}\dot{\varepsilon_{L}\;}\left(t\right)+3{\lambda_{L}}^{2}\varepsilon_{L}\left(t\right)+{\lambda_{L}}^{3}\int \varepsilon_{L}\left(t\right)dt\right]\\ \;\;\;\;\;\;+\frac{1}{n}\left[{\overset{̿}{D}}_{L}\left(t\right)+{\overset{̿}{U}}_{L}\left(t\right)\right] \end{array}$$

Furthermore, using the definition of $S_{L}(t)$ in (17), (18) can be rewritten as

(21) $${\dot{S}}_{L}=\frac{1}{n}\left[{\Delta \overset{̿}{C}}_{L}{\dot{q}}_{L}\left(t\right)+\Delta {\overset{̿}{C}}_{LS}{\dot{q}}_{s}\left(t\right)+{\overset{̿}{U}}_{L.R}\left(t\right)\right]-\lambda_{L}S_{L}+\frac{1}{n}\left[{\overset{̿}{D}}_{L}\left(t\right)+{\overset{̿}{U}}_{L}\left(t\right)\right]$$

Next, consider the following Lyapunov candidate function,

(22) $$V_{L}\left(t\right)=\frac{1}{2}{S_{L}}^{2}>0\in R$$

The derivative of $V_{L}\left(t\right)$ with respect to a time is given by

(23) $${\dot{V}}_{L}\left(t\right)=S_{L}{\dot{S}}_{L}$$

Substituting (19) into (21) yields

(24) $${\dot{V}}_{L}\left(t\right)=\left[\frac{S_{L}}{n}\left[{\Delta \overset{̿}{C}}_{L}{\dot{q}}_{L}\left(t\right)+\Delta {\overset{̿}{C}}_{LS}{\dot{q}}_{s}\left(t\right)+{\overset{̿}{U}}_{L.R}\left(t\right)\right]-\lambda_{L}{S_{L}}^{2}+\frac{S_{L}}{n}\left[{\overset{̿}{D}}_{L}\left(t\right)+{\overset{̿}{U}}_{L}\left(t\right)\right]\right]$$

Furthermore, if $\left|S_{L}\right|\geq \Phi_{L}$, applying (17) and (18) into (24) yields

(25) $$\begin{array}{c} {\dot{V}}_{L}\left(t\right)\leq \frac{S_{L}}{n}[\left[{\Delta \overset{̿}{C}}_{L}{\dot{q}}_{L}\left(t\right)+\Delta {\overset{̿}{C}}_{LS}{\dot{q}}_{s}\left(t\right)\right]-\left[\frac{k_{L}{\left|{\dot{q}}_{L}\left(t\right)\right|S}_{L}\left(t\right)}{\left|S_{L}\left(t\right)\right|}+\frac{k_{LS}{\left\|{\dot{q}}_{s}\left(t\right)\right\|S}_{L}\left(t\right)}{\left|S_{L}\left(t\right)\right|}\right]\\ +\left[{\overset{̿}{D}}_{L}\left(t\right)-\gamma_{L}\frac{S_{L}}{\left|S_{L}\right|}\right]]\\ \leq \frac{1}{n}\left[\left|S_{L}\right|\left|{\Delta \overset{̿}{C}}_{L}\right|\left|{\dot{q}}_{L}\left(t\right)\right|-\frac{k_{L}\left|{\dot{q}}_{L}\left(t\right)\right|{\left|S_{L}\left(t\right)\right|}^{2}}{\left|S_{L}\left(t\right)\right|}+\left|S_{L}\right|\left\|\Delta {\overset{̿}{C}}_{LS}\right\|\left\|{\dot{q}}_{s}\left(t\right)\right\|-\frac{k_{LS}\left\|{\dot{q}}_{s}\left(t\right)\right\|{\left|S_{L}\left(t\right)\right|}^{2}}{\left|S_{L}\left(t\right)\right|}\right]\\ +\left[\left|S_{L}\right|\left|{\;\overset{̿}{D}}_{L}\left(t\right)\right|-\gamma_{L}\frac{{\left|S_{L}\right|}^{2}}{\left|S_{L}\right|}\right]\\ \leq \frac{1}{n}\left[\left|S_{L}\right|\left[\left|{\;\overset{̿}{D}}_{L}\left(t\right)\right|-\gamma_{L}\right]\right]\leq \frac{\left|S_{L}\right|}{n}\left[{∆}_{L}-\gamma_{L}\right]\leq 0 \end{array}$$

Finally, we can see that the locked error system is ultimately bounded by (16). The proof is completed. □

**Proposition** **1.** *The time from the initial state until the system arrives* $S_{L}\left({t_{L}}^{*}\right)=\Phi_{L}$ *is also bounded by the control system (16) such that*

$${t_{L}}^{*}-t_{0}\leq \frac{n{\left(S_{L}(t_{0}\right)}^{2}-{S_{L}({t_{L}}^{*})}^{2})}{2\Phi_{L}\left[{\gamma_{L}-∆}_{L}\right]}$$

*where* $t_{0}$ *and* ${t_{L}}^{*}$ *are an initial time and a particular time when* $S_{L}\left({t_{L}}^{*}\right)=\Phi_{L}$.

**Proof.**  Based on (25), if $\left|S_{L}\right|\geq \Phi_{L}$, it is true that

(26) $${\dot{V}}_{L}\left(t\right)\leq \frac{\left|S_{L}\right|}{n}\left[{∆}_{L}-\gamma_{L}\right]$$

Integrating (26) over a time from $t_{0}$ to ${t_{L}}^{*}$ yields

(27) $$V_{L}\left({t_{L}}^{*}\right)-V_{L}\left(t_{0}\right)\leq \int_{t_{0}}^{{t_{L}}^{*}}\frac{\Phi_{L}}{n}\left[{∆}_{L}-\gamma_{L}\right]dt$$

Furthermore,

(28) $$V_{L}\left({t_{L}}^{*}\right)-V_{L}\left(t_{0}\right)\leq \frac{\Phi_{L}}{n}\left[{∆}_{L}-\gamma_{L}\right]({t_{L}}^{*}-t_{0})$$

Due to ${∆}_{L}-\gamma_{L}<0$, consequently,

(29) $${t_{L}}^{*}-t_{0}\leq \frac{n{[V}_{L}\left(t_{0}\right)-V_{L}\left({t_{L}}^{*}\right)]}{\Phi_{L}\left[{\gamma_{L}-∆}_{L}\right]}=\frac{n{\left(S_{L}(t_{0}\right)}^{2}-{S_{L}({t_{L}}^{*})}^{2})}{2\Phi_{L}\left[{\gamma_{L}-∆}_{L}\right]}$$

The duration ${t_{L}}^{*}-t_{0}$ is bounded as shown in (29). The proof is completed. □

### 4.2. Controller Design of Shape System

Similarly, this sub-section presents the controller design of the shape system and shows the stability of the proposed controller via the Lyapunov approach.

Revisiting (14),

(30) $${\Upsilon \ddot{q}}_{S}\left(t\right)=-\left[{\overset{̿}{C}}_{S}{\dot{q}}_{S}\left(t\right)+{\overset{̿}{C}}_{SL}{\dot{q}}_{L}\left(t\right)\right]+{\overset{̿}{T}}_{S}\left(t\right)+{\overset{̿}{D}}_{S}\left(t\right)$$

Multiplying *n* to both sides of (30),

(31) $${n\Upsilon \ddot{q}}_{S}\left(t\right)=\left[-n\left[{\overset{̿}{C}}_{S}{\dot{q}}_{S}\left(t\right)+{\overset{̿}{C}}_{SL}{\dot{q}}_{L}\left(t\right)\right]+n\left[{\overset{̿}{T}}_{S}\left(t\right)+{\overset{̿}{D}}_{S}\left(t\right)\right]\right]$$

Due to the fact that ${n\Upsilon \ddot{q}}_{S}\left(t\right)={\ddot{q}}_{S}\left(t\right)$, (31) becomes

(32) $${\ddot{q}}_{S}\left(t\right)=n\left[-{\overset{̿}{C}}_{S}{\dot{q}}_{S}\left(t\right)-{\overset{̿}{C}}_{SL}{\dot{q}}_{L}\left(t\right)+{\overset{̿}{T}}_{S}\left(t\right)+{\overset{̿}{D}}_{S}\left(t\right)\right]$$

Proposing the control law for (32),

(33) $$\begin{array}{c} {\overset{̿}{T}}_{S}\left(t\right)=\tilde{{\overset{̿}{C}}_{S}}{\dot{q}}_{S}\left(t\right)+\tilde{{\overset{̿}{C}}_{SL}}{\dot{q}}_{L}\left(t\right)-\frac{1}{n}\left[3\lambda_{S}\dot{q_{S}\;}\left(t\right)+3{\lambda_{S}}^{2}q_{S}\left(t\right)+{\lambda_{S}}^{3}\int q_{S}\left(t\right)dt\right]\\ +{\overset{̿}{U}}_{S.R}\left(t\right)+{\overset{̿}{U}}_{S}\left(t\right) \end{array}$$

(34) $${\overset{̿}{U}}_{S.R}\left(t\right)=\left\{\begin{array}{c} -\frac{k_{S}\left\|{\dot{q}}_{S}\left(t\right)\right\|S_{S}(t)}{\left\|S_{S}(t)\right\|}-\frac{k_{SL}\left|{\dot{q}}_{L}\left(t\right)\right|S_{S}(t)}{\left\|S_{S}(t)\right\|}\\ 0 \end{array}\right.\begin{array}{c} \;\;if\left\|S_{S}(t)\right\|\geq \Phi_{S}\\ \;\;\;if\left\|S_{S}(t)\right\|<\Phi_{S} \end{array}$$

(35) $${\overset{̿}{U}}_{S}\left(t\right)=\left\{\begin{array}{c} -\gamma_{S}S_{s}(t)/\left\|S_{s}(t)\right\|\\ -\gamma_{S}S_{s}(t)/\Phi_{S} \end{array}\right.\begin{array}{c} \;\;if\left\|S_{S}(t)\right\|\geq \Phi_{S}\\ \;\;if\left\|S_{S}(t)\right\|<\Phi_{S} \end{array}$$

(36) $$S_{S}(t)=\dot{q_{S}\;}\left(t\right)+2\lambda_{S}q_{S}\left(t\right)+{\lambda_{S}}^{2}\int q_{S}\left(t\right)dt\in R^{(p-1)\times 1}$$

where $\lambda_{S}=daig\left[\begin{array}{ccc} \begin{array}{cc} \lambda_{S.1} & \lambda_{S.2} \end{array} & \cdots & \lambda_{S.(p-1)} \end{array}\right]\in R^{\left(p-1)\times (p-1\right)}$ is the control gain matrix and $S_{S}(t)\in R^{(p-1)\times 1}$ is the sliding surface, which is a function of the shape coordinate $q_{S}\left(t\right)$. Similar to the previous case, $\tilde{{\overset{̿}{C}}_{S}}$ and $\tilde{{\overset{̿}{C}}_{SL}}$ are the nominal values of ${\overset{̿}{C}}_{S}$ and ${\overset{̿}{C}}_{SL}$ and it is assumed that $\left|\tilde{{\overset{̿}{C}}_{S}}-{\overset{̿}{C}}_{S}\right|\leq k_{S}$ and $\left\|\tilde{{\overset{̿}{C}}_{SL}}-{\overset{̿}{C}}_{SL}\right\|\leq k_{SL}$ with $k_{S}\in R>0$ and $k_{SL}\in R>0$. Also, $\Phi_{S}$ ∈R > 0 is the thickness of the boundary layer. Similar to (16), for the shape system, the compensating term in (34) rejects the model uncertainty and (35) is used to dismantle the external disturbance.

**Theorem** **2.** *As long as the parametric uncertainty and external disturbance is bounded such as $\left\|\Delta {\overset{̿}{C}}_{S}\right\|\leq k_{S}$, $\left\|\Delta {\overset{̿}{C}}_{SL}\right\|\leq k_{SL}$ and $\left\|{\overset{̿}{D}}_{S}\left(t\right)\right\|\leq {∆}_{S}\in R$ with $\gamma_{S}>{∆}_{S}$, the shape system (32) is ultimately bounded by the controller in (33), where $\Delta {\overset{̿}{C}}_{S}=\tilde{{\overset{̿}{C}}_{S}}-{\overset{̿}{C}}_{S}$ and $\Delta {\overset{̿}{C}}_{SL}=\tilde{{\overset{̿}{C}}_{SL}}-{\overset{̿}{C}}_{SL}$.*

**Proof.**  Substituting (33) into (32), the shape closed-loop system is given by

(37) $$\begin{array}{c} {\ddot{q}}_{S}\left(t\right)=n\left[\Delta {\overset{̿}{C}}_{S}{\dot{q}}_{S}\left(t\right)+\Delta {\overset{̿}{C}}_{SL}{\dot{q}}_{L}\left(t\right)+{\overset{̿}{U}}_{S.R}\left(t\right)\right]-\left[3\lambda_{S}\dot{q_{S}\;}\left(t\right)+3{\lambda_{S}}^{2}q_{S}\left(t\right)+{\lambda_{S}}^{3}\int q_{S}\left(t\right)dt\right]\\ \;\;\;\;\;\;\;+n\left[{\overset{̿}{D}}_{S}\left(t\right)+{\overset{̿}{U}}_{S}\left(t\right)\right] \end{array}$$

Furthermore, (37) becomes

(38) $${\dot{S}}_{S}=n\left[\Delta {\overset{̿}{C}}_{S}{\dot{q}}_{S}\left(t\right)+\Delta {\overset{̿}{C}}_{SL}{\dot{q}}_{L}\left(t\right)+{\overset{̿}{U}}_{S.R}\left(t\right)\right]-\lambda_{S}S_{S}+n\left[{\overset{̿}{D}}_{S}\left(t\right)+{\overset{̿}{U}}_{S}\left(t\right)\right]$$

Similarly, consider the following Lyapunov candidate function,

(39) $$V_{s}\left(t\right)=\frac{1}{2}{S_{S}}^{T}S_{S}>0\in R$$

The derivative of $V_{S}\left(t\right)$ with respect to a time is given by

(40) $${\dot{V}}_{S}\left(t\right)={S_{S}}^{T}{\dot{S}}_{S}$$

Substituting (38) into (40) yields

(41) $$\begin{array}{l} {\dot{V}}_{S}\left(t\right)={S_{S}}^{T}\left[n\left[\Delta {\overset{̿}{C}}_{S}{\dot{q}}_{S}\left(t\right)+\Delta {\overset{̿}{C}}_{SL}{\dot{q}}_{L}\left(t\right)+{\overset{̿}{U}}_{S.R}\left(t\right)\right]-\lambda_{L}S_{S}+n\left[{\overset{̿}{D}}_{S}\left(t\right)+{\overset{̿}{U}}_{S}\left(t\right)\right]\right]\\ \;=\left[n{S_{S}}^{T}\left[\Delta {\overset{̿}{C}}_{S}{\dot{q}}_{S}\left(t\right)+\Delta {\overset{̿}{C}}_{SL}\Delta {\dot{q}}_{L}\left(t\right)+{\overset{̿}{U}}_{S.R}\left(t\right)\right]-\lambda_{L}{S_{S}}^{T}S_{S}+n{S_{S}}^{T}\left[{\overset{̿}{D}}_{S}\left(t\right)+{\overset{̿}{U}}_{S}\left(t\right)\right]\right] \end{array}$$

Furthermore, if $\left\|S_{S}\right\|\geq \Phi_{S}$, applying (34) and (35) into (41) yields

(42) $$\begin{array}{c} {\dot{V}}_{S}\left(t\right)\leq n{S_{S}}^{T}\left[\Delta {\overset{̿}{C}}_{S}{\dot{q}}_{S}\left(t\right)+\Delta {\overset{̿}{C}}_{SL}{\dot{q}}_{L}\left(t\right)+{\overset{̿}{U}}_{S.R}\left(t\right)\right]+n{S_{S}}^{T}\left[{\overset{̿}{D}}_{S}\left(t\right)+{\overset{̿}{U}}_{S}\left(t\right)\right]\\ \leq n\left\|S_{S}\right\|\left\|\Delta {\overset{̿}{C}}_{S}\right\|\left\|{\dot{q}}_{S}\left(t\right)\right\|-n\frac{k_{S}\left\|{\dot{q}}_{S}\left(t\right)\right\|{{S_{S}}^{T}S}_{S}}{\left\|S_{S}\left(t\right)\right\|}+n\left\|S_{S}\right\|\left\|\Delta {\overset{̿}{C}}_{SL}\right\|\left|{\dot{q}}_{L}\left(t\right)\right|-n\frac{k_{SL}\left|{\dot{q}}_{L}\left(t\right)\right|{{S_{S}}^{T}S}_{S}}{\left\|S_{S}(t)\right\|}\\ +n\left[\left\|S_{S}\right\|\left\|{\overset{̿}{D}}_{S}\left(t\right)\right\|-\frac{\gamma_{S}{{S_{S}}^{T}S}_{s}}{\left\|S_{s}\right\|}\right]\\ \leq n\left\|S_{S}\right\|\left\|\Delta {\overset{̿}{C}}_{S}\right\|\left\|{\dot{q}}_{S}\left(t\right)\right\|-nk_{S}\left\|{\dot{q}}_{S}\left(t\right)\right\|\left\|S_{S}\left(t\right)\right\|+n\left\|S_{S}\right\|\left\|\Delta {\overset{̿}{C}}_{SL}\right\|\left|{\dot{q}}_{L}\left(t\right)\right|\\ -nk_{SL}\left|{\dot{q}}_{L}\left(t\right)\right|\left\|S_{S}(t)\right\|+n\left[\left\|S_{S}\right\|\left\|{\overset{̿}{D}}_{S}\left(t\right)\right\|-\frac{\gamma_{S}{{S_{S}}^{T}S}_{s}}{\left\|S_{s}\right\|}\right]\\ \leq n\left[\left\|S_{S}\right\|\left\|{\overset{̿}{D}}_{S}\left(t\right)\right\|-\frac{\gamma_{S}{\left\|S_{s}\right\|}^{2}}{\left\|S_{s}\right\|}\right]\leq n\left\|S_{S}\right\|\left[{∆}_{S}-\gamma_{s}\right]<0 \end{array}$$

Finally, we can see that the shape system is ultimately bounded by (33). The proof is completed. □

**Proposition** **2.** *The time from the initial state until the system reaches* $S_{S}\left({t_{S}}^{*}\right)=\Phi_{S}$ *is also bounded by the control system (33) as*

$${t_{S}}^{*}-t_{0}\leq \frac{{\left(S_{S}(t_{0}\right)}^{T}S_{S}(t_{0})-{S_{S}({t_{S}}^{*})}^{T}S_{S}({t_{S}}^{*}))}{2n\Phi_{S}\left[{\gamma_{s}-∆}_{s}\right]}$$

*where* $t_{0}$ *and* ${t_{S}}^{*}$ *are the initial time and a particular time for* $S_{S}\left({t_{S}}^{*}\right)=\Phi_{S}$.

**Proof.**  Based on (42), if $\left\|S_{S}\right\|>\Phi_{S}$, it is clear that

(43) $${\dot{V}}_{S}\left(t\right)\leq n\left\|S_{S}\right\|\left[{∆}_{S}-\gamma_{s}\right]$$

Integrating (43) over a time from $t_{0}$ to $t^{*}$ yields

(44) $$V_{S}\left({t_{S}}^{*}\right)-V_{S}\left(t_{0}\right)\leq \int_{t_{0}}^{{t_{S}}^{*}}n\Phi_{S}\left[{∆}_{S}-\gamma_{s}\right]dt$$

Consequently,

(45) $${t_{S}}^{*}-t_{0}\leq \frac{V_{S}\left(t_{0}\right)-V_{S}\left({t_{S}}^{*}\right)}{{n\Phi }_{L}\left[{\gamma_{s}-∆}_{s}\right]}=\frac{{\left(S_{S}(t_{0}\right)}^{T}S_{S}(t_{0})-{S_{S}({t_{S}}^{*})}^{T}S_{S}({t_{S}}^{*}))}{2n\Phi_{S}\left[{\gamma_{s}-∆}_{s}\right]}$$

The duration ${t_{S}}^{*}-t_{0}$ is bounded as shown in (45). The proof is complete. □

**Remark** **2.** *Both control systems according to locked and shape coordinates are designed as shown in (16) and (33). It should be stated that the actual control torque of motors in physical coordinate can be obtained by the following transformation,*

(46) $$\begin{array}{c} T\left(t\right)={M\left(\Gamma \Gamma^{T}\right)}^{-1}\Gamma \overset{̿}{T}\left(t\right)\\ {\left[\tau_{i}\right]}^{T}={M\left(\Gamma \Gamma^{T}\right)}^{-1}\Gamma {\left[\begin{array}{cc} {\overset{̿}{T}}_{L}\left(t\right) & {{\overset{̿}{T}}_{S}\left(t\right)}^{T} \end{array}\right]}^{T}for\;\;i=1,2,\cdots n \end{array}$$

## 5. Other Synchronous Control Approaches

**Figure 2:**
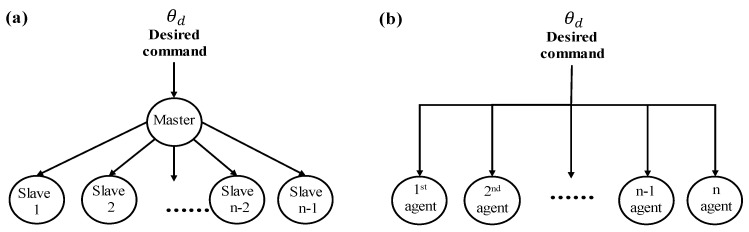
Synchronous control approaches. (**a**) Master–slave control approach and (**b**) independent control approach.

To perform the comparison study, this section presents the other two well-known synchronous controls. The first approach is a master–slave control system while the second one is an independent control system. The configurations of both control systems are briefly described in Figure 2.

For the master–slave control in Figure 2a, the desired command is delivered to the master, one of agents, and the slaves (the rest of agents) receive the master’s command. On the other hand, an independent control forces all agents to individually receive the desired command as shown in Figure 2b. The control performance of these two will be compared with the performance of the proposed control in Section 4.

### 5.1. Master–Slave Control Approach

The control laws for a master and slave scheme to ensure robust synchronous stability of agents are contained in this sub-section.

Proposing the control law for a master,

(47) $$\begin{array}{c} \tau_{m}=J_{m,m}{\ddot{\theta }}_{d}+{\hat{B}}_{m,m}{\dot{\theta }}_{m}-J_{m,m}\left(3\lambda_{m}{\dot{\varepsilon }}_{m}+3\lambda_{m}^{2}\varepsilon_{m}+\lambda_{m}^{3}\int \varepsilon_{m}dt\right)+u_{m..R}+u_{m}\\ u_{m.R}=\left\{\begin{array}{c} -k_{m}\left|{\dot{\theta }}_{m}\right|sign\left(S_{m}\right)\;\;\;\;\;\;if\;\left|S_{m}\right|>N_{m}\\ 0\;\;\;\;\;\;\;\;\;\;\;\;\;\;\;\;\;\;\;\;\;\;\;\;\;\;\;\;\;\;\;\;\;\;\;if\;\left|S_{m}\right|\leq N_{m} \end{array}\right.\;\;\;\;\;u_{m}=\left\{\begin{array}{c} -H_{m}sign\left(S_{m}\right)\;\;\;\;\;\;if\;\left|S_{m}\right|>N_{m}\\ -H_{m}\frac{S_{m}}{N_{m}}\;\;\;\;\;\;\;\;\;\;\;\;\;\;\;\;if\;\left|S_{m}\right|\leq N_{m} \end{array}\right. \end{array}$$

where the error $\varepsilon_{m}=\theta_{m}-\theta_{d}$ and a sliding surface $S_{m}={\dot{\varepsilon }}_{m}+2\lambda_{m}\varepsilon_{m}+\lambda_{m}^{2}\int \varepsilon_{m}dt$. And, ${\hat{B}}_{m,m}$ is the nominal value of $B_{m.1}$ and satisfies $\left|{\hat{B}}_{m,m}-B_{m.1}\right|<k_{m}$.

The control laws for the slaves are given by,

(48) $$\begin{array}{c} \tau_{j}=J_{m,j}{\ddot{\theta }}_{m}+{\hat{B}}_{m,j}{\dot{\theta }}_{j}-J_{m,j}\left(3\lambda_{j}{\dot{\varepsilon }}_{j}+3\lambda_{j}^{2}\varepsilon_{j}+\lambda_{j}^{3}\int \varepsilon_{j}\right)+u_{j.R}+u_{j}\;\;\mathrm{for}\;j=1,2,\cdots n-1\\ u_{j.R}=\left\{\begin{array}{c} -k_{.j}\left|{\dot{\theta }}_{j}\right|sign\left(S_{j}\right)\;\;\;\;\;\;if\;\left|S_{j}\right|>N_{j}\\ 0\;\;\;\;\;\;\;\;\;if\;\left|S_{j}\right|\leq N_{j} \end{array}\right.\;\;\;\;\;\;u_{j}=\left\{\begin{array}{c} -H_{j}sign\left(S_{j}\right)\;\;\;\;\;\;if\;\left|S_{j}\right|>N_{j}\\ -H_{j}\frac{S_{j}}{N_{j}}\;\;\;\;\;\;\;\;\;\;if\;\left|S_{j}\right|\leq N_{j} \end{array}\right. \end{array}$$

where $\varepsilon_{j}=\theta_{j}-\theta_{m}$ for j=1,2,⋯n−1 is the error and $S_{j}={\dot{\varepsilon }}_{j}+2\lambda_{j}\varepsilon_{j}+\lambda_{j}^{2}\int \varepsilon_{j}dt$ is the sliding surface. By using both (47) and (48), $\theta_{j}\to \theta_{m}\to \theta_{d}$ can be achieved and the corresponding stability proof is omitted due to simplicity.

### 5.2. Independent Control Approach

This sub-section details an independent control law for the individual agents.

The control law for each motor under an independent control approach is followed by

(49) $$\begin{array}{c} \tau_{i}=J_{m,i}{\ddot{\theta }}_{d}+{\hat{B}}_{m,i}{\dot{\theta }}_{i}-J_{m,i}\left(3\lambda_{i}{\dot{\varepsilon }}_{i}+3\lambda_{i}^{2}\varepsilon_{i}+\lambda_{i}^{3}\int \varepsilon_{i}\right)+u_{i.R}+u_{i}\;\mathrm{for}\;i=1,2,3,\cdots n\\ u_{i.R}=\left\{\begin{array}{c} -k_{i}\left|{\dot{\theta }}_{i}\right|sign\left(S_{i}\right)\;\;\;\;\;\;if\;\left|S_{i}\right|>N_{i}\\ \;\;0\;\;\;\;\;\;\;\;\;if\;\left|S_{i}\right|\leq N_{i} \end{array}\right.\;\;\;\;\;\;\;\;u_{i}=\left\{\begin{array}{c} -H_{i}sign\left(S_{i}\right)\;\;\;\;\;\;if\;\left|S_{i}\right|>N_{i}\\ \;-H_{i}\frac{S_{i}}{N_{i}}\;\;\;\;\;\;\;\;\;if\;\left|S_{i}\right|\leq N_{i} \end{array}\right. \end{array}$$

where $\varepsilon_{i}=\theta_{i}-\theta_{d}$ for i=1,2,3,⋯n is the error and $S_{i}={\dot{\varepsilon }}_{i}+2\lambda_{i}\varepsilon_{i}+\lambda_{i}^{2}\int \varepsilon_{i}dt$ is the sliding surface.

The above control can achieve $\theta_{i}\to \theta_{d}$ and the corresponding stability proof is omitted for simplicity.

## 6. Experimental Study and Results

This section validates the effectiveness of the proposed synchronous control strategy via experimental studies on two BLDC motors, three BLDC motors, and four BLDC motors. The BLDC motors used here are the cost-effective ones featured with a high dead-zone property, which is difficult to precisely control. The reason why we selected such motors is that we desired to demonstrate that the control performance of our proposed control is effective and robust. In addition, the control performance has been compared with the other approaches presented in Section 5.

Before proceeding, the transformation matrices from the physical coordinate to the locked-shape one for the two agents, the three agents, and the four agents are introduced based on (6) for a clear understanding of proposed control system.

For the two motors,

(50) $$H=\left[\begin{array}{cc} 1/2 & 1/2\\ 1 & -1 \end{array}\right]\in R^{2\times 2}$$

For the three motors,

(51) $$H=\left[\begin{array}{ccc} 1/3 & 1/3 & 1/3\\ 1 & -1 & 0\\ \begin{array}{c} 1\\ 0 \end{array} & \begin{array}{c} 0\\ 1 \end{array} & \begin{array}{c} -1\\ -1 \end{array} \end{array}\right]\in R^{4\times 3}$$

For the four motors,

(52) $$H=\left[\begin{array}{ccc} 1/4 & 1/4 & \begin{array}{cc} 1/4 & 1/4 \end{array}\\ 1 & -1 & \begin{array}{cc} 0 & 0 \end{array}\\ \begin{array}{c} 1\\ \begin{array}{c} 1\\ 0\\ \begin{array}{c} 0\\ 0 \end{array} \end{array} \end{array} & \begin{array}{c} 0\\ \begin{array}{c} 0\\ 1\\ \begin{array}{c} 1\\ 0 \end{array} \end{array} \end{array} & \begin{array}{c} \begin{array}{cc} -1 & 0 \end{array}\\ \begin{array}{cc} \begin{array}{c} 0\\ -1\\ \begin{array}{c} 0\\ 1 \end{array} \end{array} & \begin{array}{c} -1\\ 0\\ \begin{array}{c} -1\\ -1 \end{array} \end{array} \end{array} \end{array} \end{array}\right]\in R^{7\times 4}$$

In addition, the main control gains ($\lambda_{L}$ and $\lambda_{S}$) of (16) and (33) were selected as 34 and 32, respectively, and are used for every experiment. Here, the selection of these gains was determined by the trial and error method, but it can be explored theoretically in a future study.

### 6.1. Experimental Results of Two Agents

**Figure 3:**
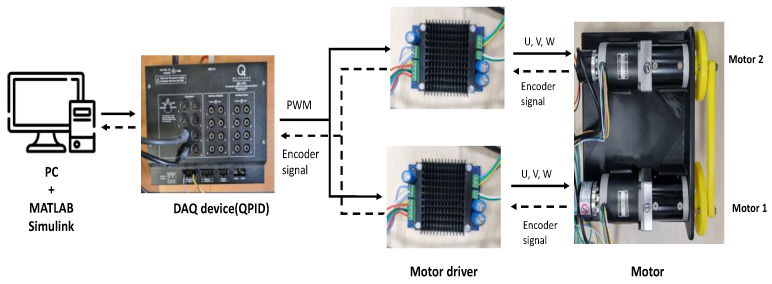
Experimental setup for two BLDC motors.

Figure 3 includes the experimental setup for the synchronous control of two motors. Two 12 V geared BLDC motors (gear ratio 16:1) and two BLDC motor drivers are displayed and connected to a DAQ (QPID) that communicates with PC/MATLAB, where the control strategy is implemented with a sampling time of 0.005 s. Also, each motor is equipped with a rotational encoder to provide the angular position of the motor. To increase the external disturbance, two motors are interconnected with the bar jointing of two rotors of motors (see Figure 3). The synchronous control of this dual-motor driving setting shown in Figure 3 can be applied to a dual-motor driving steering system in a vehicle.

**Figure 4:**
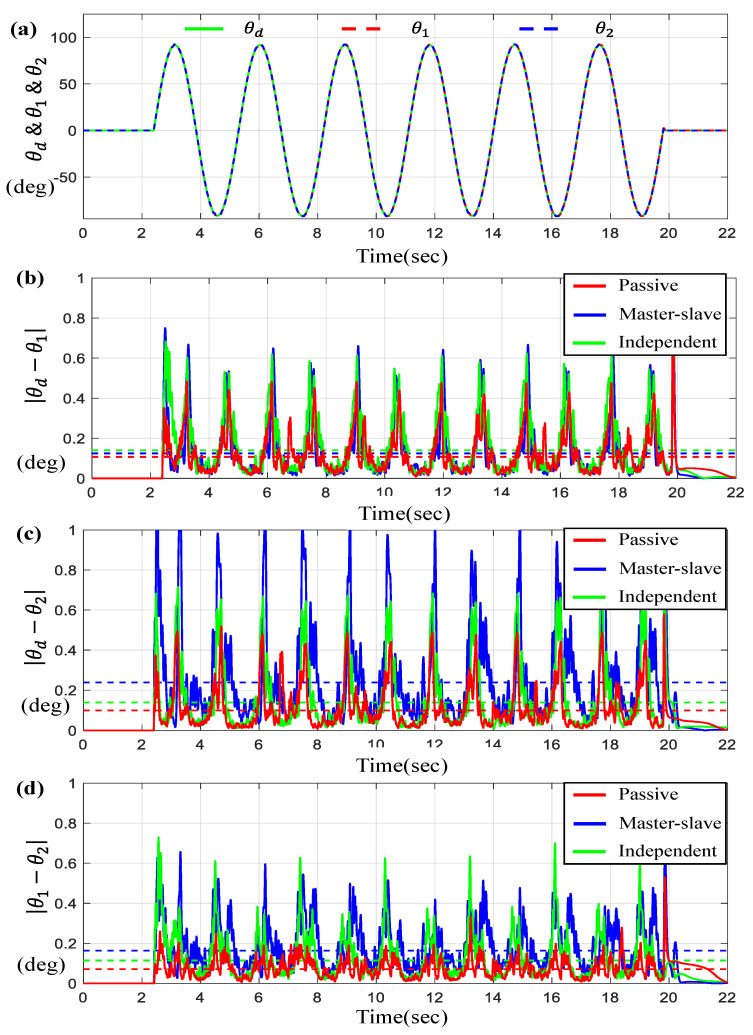
Results for two BLDC motors under sine-wave trajectory. (**a**) $\theta_{d}$, $\theta_{1}$, and $\theta_{2}$ on the time-domain, (**b**) $\left|\theta_{d}-\theta_{1}\right|$, (**c**) $\left|\theta_{d}-\theta_{2}\right|$, and (**d**) $\left|\theta_{1}-\theta_{2}\right|$ (the dotted lines indicate the average values).

For the scenario of the sine-wave trajectory (denoted as $\theta_{d}$) with the maximum amplitude of 90 deg. and a frequency of 1 Hz, Figure 4 described the tracking performance and synchronous error of each motor, $\theta_{1}$ and $\theta_{2},$ for a given $\theta_{d}$. Figure 4a represent $\theta_{1}$ and $\theta_{2}$ along with $\theta_{d}$ on the time-domain and Figure 4b,c represent the tracking errors $\left|\theta_{i}-\theta_{d}\right|$ for *i* = 1, 2. Figure 4d depicts the synchronous error $\left|\theta_{1}-\theta_{2}\right|$. Here, the dotted lines shown in Figure 4b–d indicate the average tracking errors. We can see that the maximum and average tracking errors via the proposed controller are approximately 0.8 degree and 0.1 degree, and the corresponding synchronous error is bounded below 0.6 degree. Also, it is found from the results that the average tracking errors and the maximum errors generated by the proposed controller (denoted as “passive”) are the smallest among the three controllers. Table 1 contains the numerical values for the average tracking and the maximum errors via three controllers for the sine-wave trajectory. Specifically, it is found that the maximum and average errors of the proposed controller are 25%~45% smaller than those of other control schemes.

**Figure 5:**
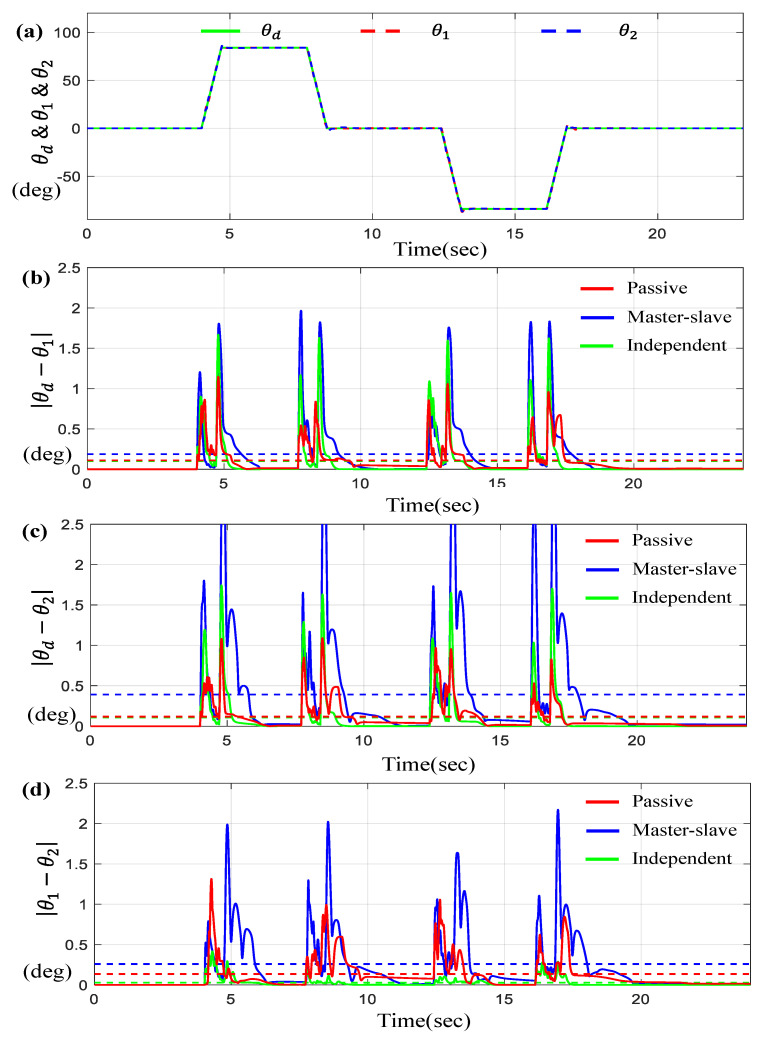
Results for two BLDC motors under trapezoidal trajectory (**a**) $\theta_{d}$, $\theta_{1}$, and $\theta_{2}$ on the time-domain, (**b**) $\left|\theta_{d}-\theta_{1}\right|$, (**c**) $\left|\theta_{d}-\theta_{2}\right|$, and (**d**) $\left|\theta_{1}-\theta_{2}\right|$ (the dotted lines indicate the average values).

On the other hand, Figure 5 presents the tracking and synchronous performance of motors, for the trapezoidal trajectory with the maximum amplitude of 90 deg. and slope of 90 deg/s.

Similar to Figure 4, Figure 5a represents $\theta_{1}$ and $\theta_{2}$ with a given $\theta_{d}$ on the time-domain. Figure 5b,c indicate the tracking errors $\left|\theta_{i}-\theta_{d}\right|$ for *i* = 1, 2 while Figure 5d describes the synchronous error $\left|\theta_{1}-\theta_{2}\right|$. It can be seen from the results that the tracking and synchronous errors of the proposed control for this scenario are bounded below 1.3 degree.

Unlike the results in Figure 4, the independent control method is superior to other methods, but slightly better than the proposed method. It is clear that the performance difference between “passive” and “independent” are almost equivalent for each other except for the synchronous error $\left|\theta_{1}-\theta_{2}\right|$ shown in Figure 5d.

And, for this case, the outcome via the proposed controller is definitively much better than the ones via the master–slave control approach. Table 2 lists the numerical values for the average tracking and the maximum errors via three controllers for trapezoidal trajectory tracking.

According to Table 2, the maximum synchronous error $\left|\theta_{1}-\theta_{2}\right|$ of the proposed control is 45% greater than that of the independent control, but the rest of the results are very similar for each other within about 10% difference.

### 6.2. Experimental Results of Three Agents

**Figure 6:**
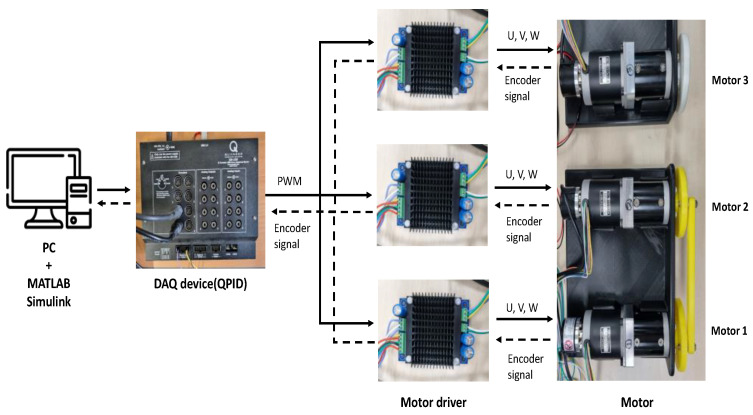
Experimental setup for three BLDC motors.

Figure 6 presents the experimental setup for the synchronous control of three motors. Similar to the case of two motors, to increase the external disturbance, two motors are connected with the bar jointing of two rotors of motors, but the last motor is free from the connection (see Figure 6). The control of the triple-motor driving setting shown in Figure 4 can be applied to a synchronous pitch angle control of three blades in a wind-turbine system.

**Figure 7:**
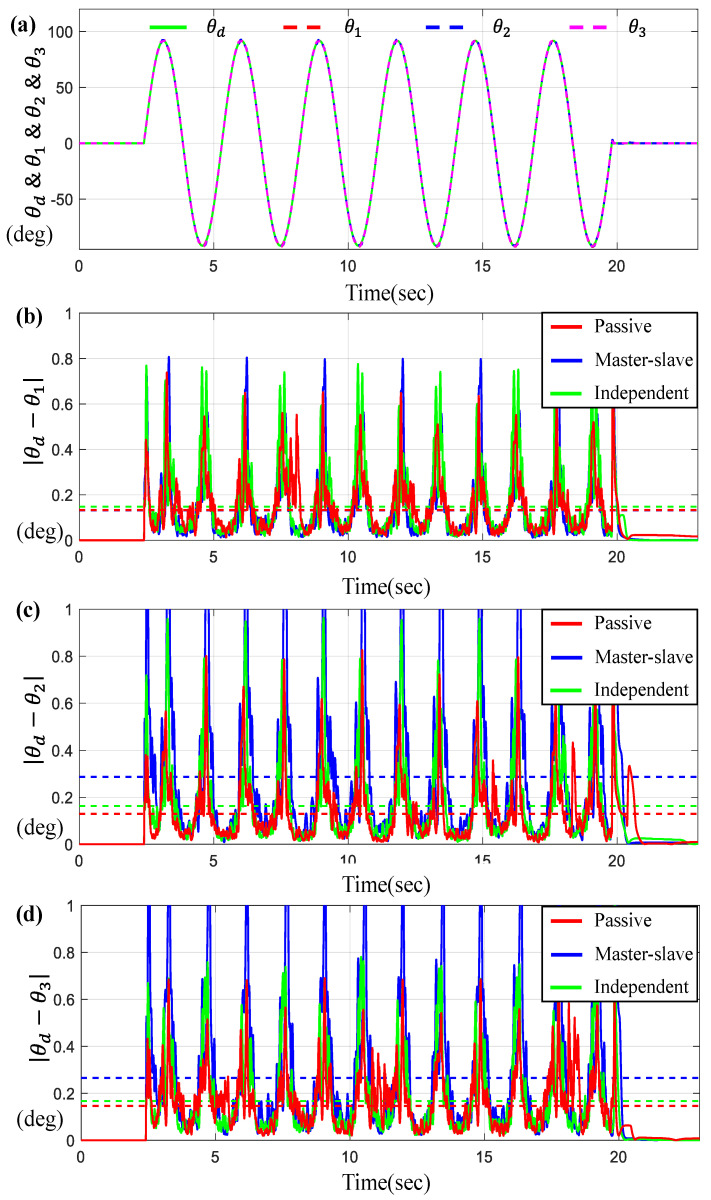
Results for three BLDC motors under sine-wave trajectory. (**a**) $\theta_{d}$, $\theta_{1}$, $\;\theta_{2}$ and $\theta_{3}$ on the time-domain, (**b**) Error $\left|\theta_{d}-\theta_{1}\right|$, (**c**) Error $\left|\theta_{d}-\theta_{2}\right|$, and (**d**) Error $\left|\theta_{d}-\theta_{3}\right|$ (the dotted lines indicate the average values).

**Figure 8:**
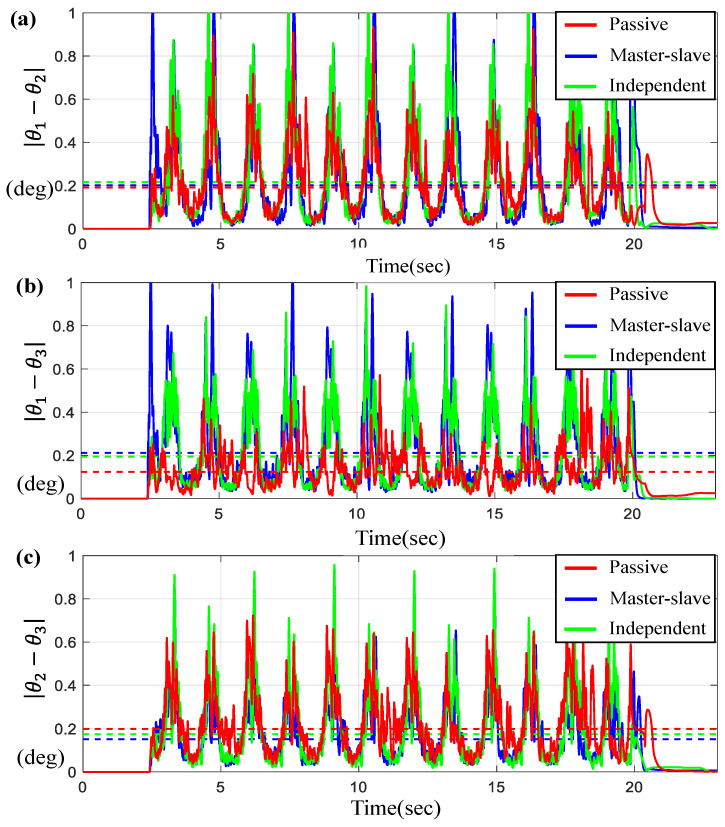
Results for three BLDC motors under sine-wave trajectory. (**a**) Error $\left|\theta_{1}-\theta_{2}\right|$, (**b**) Error $\left|\theta_{1}-\theta_{3}\right|$, and (**c**) Error $\left|\theta_{2}-\theta_{3}\right|$ (the dotted lines indicate the average values).

For the scenario of the sine-wave trajectory, Figure 7 and Figure 8 describe the tracking and synchronous performance of motors, $\theta_{1}$, $\theta_{2},$ and $\theta_{3}$.

Figure 7a represents $\theta_{1}$, $\theta_{2},$ and $\theta_{3}$ along with $\theta_{d}$ on the time-domain and Figure 7b–d display the tracking errors $\left|\theta_{i}-\theta_{d}\right|$ for *i* = 1, 2, 3. Here, the dotted lines shown in Figure 7b–d indicate the average tracking errors. On the other hand, Figure 8 depicts the synchronous errors $\left|\theta_{1}-\theta_{2}\right|$, $\left|\theta_{1}-\theta_{3}\right|$ and $\left|\theta_{2}-\theta_{3}\right|$. From the results in Figure 7 and Figure 8, we can see that the average tracking errors and the maximum errors generated by the proposed controller are the smallest among the three controllers except in two cases, $\left|\theta_{1}-\theta_{d}\right|$ and $\left|\theta_{2}-\theta_{3}\right|$ (shown in Figure 8c). However, those two cases exhibit small gaps relative to the results obtained by other control approaches. It is also found that the tracking and synchronous errors of the proposed control for this case are bounded below 1 degree. Table 3 includes the numerical values for the average tracking and the maximum errors via three controllers under the sine-wave trajectory. As seen from Table 3, the errors generated by the proposed control system are approximately 10~35% less than the errors via others.

**Figure 9:**
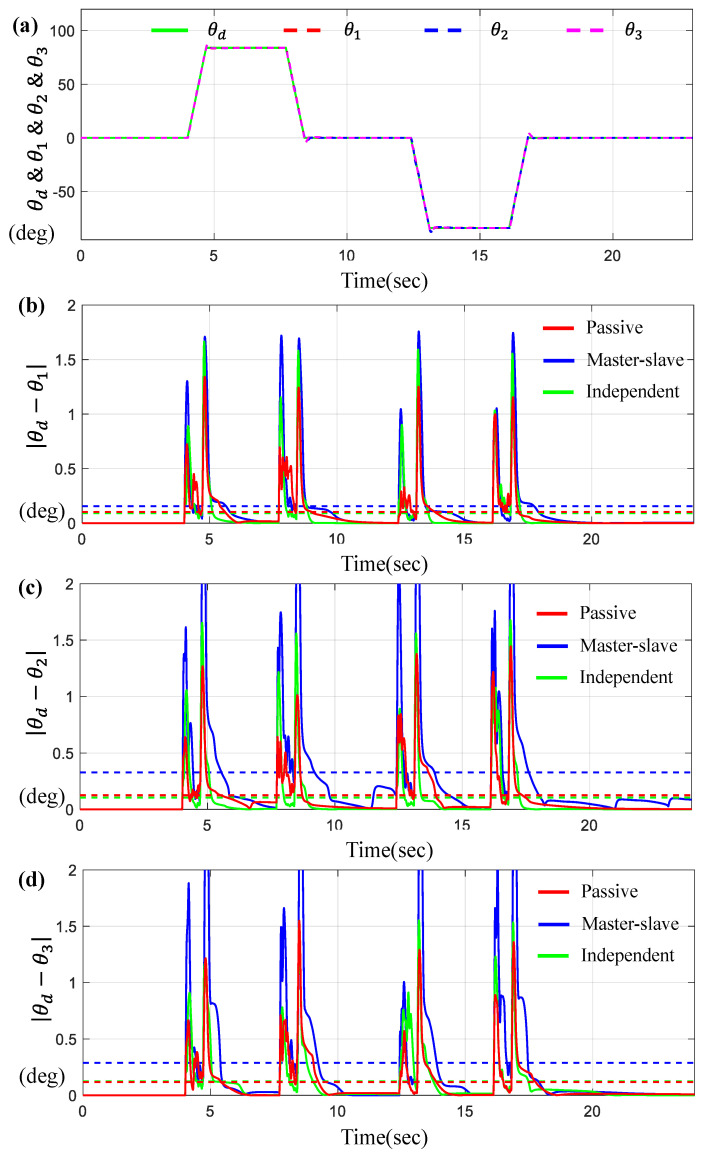
Results for three BLDC motors under trapezoidal trajectory. (**a**) $\theta_{d}$, $\theta_{1}$, $\;\theta_{2}$ and $\theta_{3}$ on the time-domain, (**b**) Error $\left|\theta_{d}-\theta_{1}\right|$, (**c**) Error $\left|\theta_{d}-\theta_{2}\right|$, and (**d**) Error $\left|\theta_{d}-\theta_{3}\right|$ (the dotted lines indicate the average values).

Furthermore, Figure 9 presents the tracking and synchronous errors of motors for the trapezoidal trajectory.

**Figure 10:**
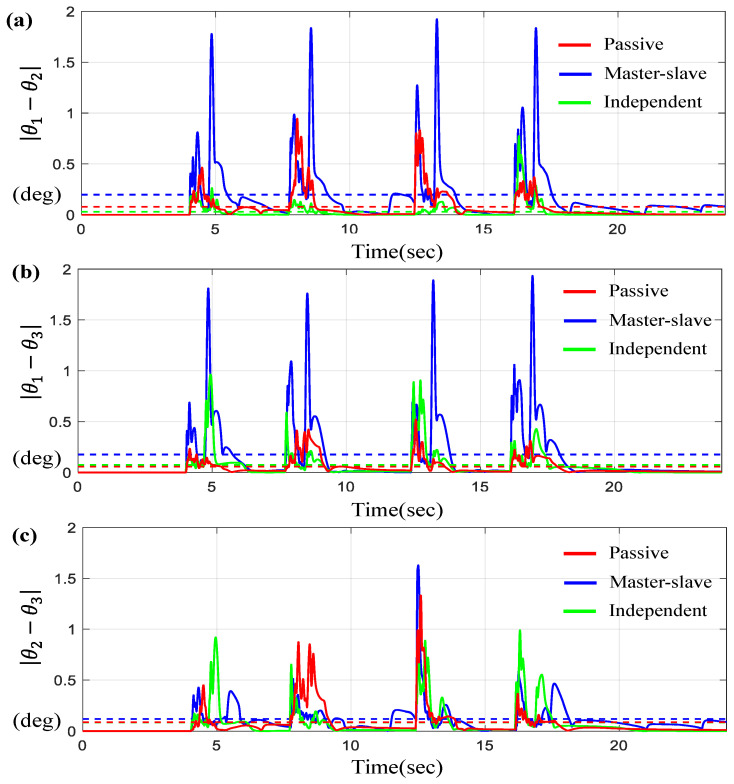
Results for three BLDC motors under trapezoidal trajectory. (**a**) Error $\left|\theta_{1}-\theta_{2}\right|$, (**b**) Error $\left|\theta_{1}-\theta_{3}\right|$, and (**c**) Error $\left|\theta_{2}-\theta_{3}\right|$ (the dotted lines indicate the average values).

Figure 9a indicates $\theta_{1}$, $\theta_{2},$ and $\theta_{3}$ for a given $\theta_{d}$ on the time-domain. Figure 9b–d show the tracking errors $\left|\theta_{i}-\theta_{d}\right|$ for *i* = 1, 2 while Figure 10 describes the synchronous errors, $\left|\theta_{1}-\theta_{2}\right|$, $\left|\theta_{2}-\theta_{3}\right|$, and $\left|\theta_{1}-\theta_{3}\right|$. Here, you can see that the tracking and synchronous errors via the proposed control are bounded below 1.5 degree.

Similar to the results in the previous cases (two motors), the independent control method is slightly superior to other methods, but is almost equivalent to our proposed method.

And, again, it is apparent that the proposed controller is definitively dominant to the master–slave control approach. Table 4 lists the numerical values of the average tracking and the maximum errors via three controllers under trapezoidal trajectory, and we can find a good match between “passive” and “independent” controls within 10%.

### 6.3. Experimental Results of Four Agents

**Figure 11:**
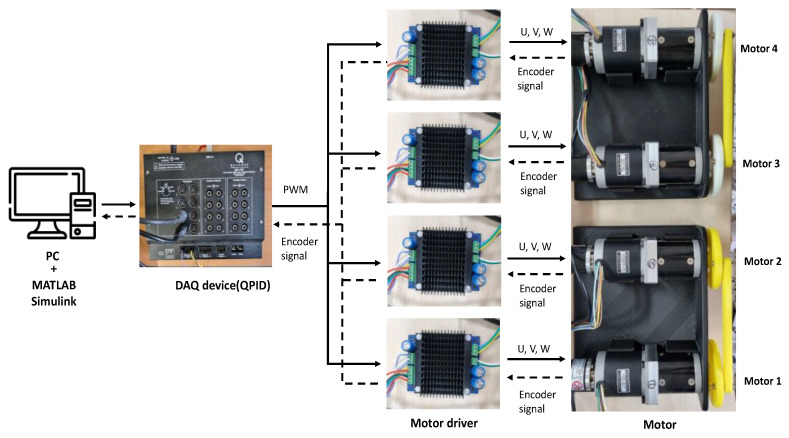
Experimental setup for four BLDC motors.

Figure 11 shows the experimental setup to validate the synchronous control performance of four motors. In this case, the 1st and 2nd motors are coupled with a rod to increase asynchronous external perturbations and the 3rd and 4th motors were connected in the same way (see Figure 11). The control of the quadruplet-motor driving setting shown in Figure 5 can be the fundamental study for a synchronous control of four independent steering controls in a vehicle.

**Figure 12:**
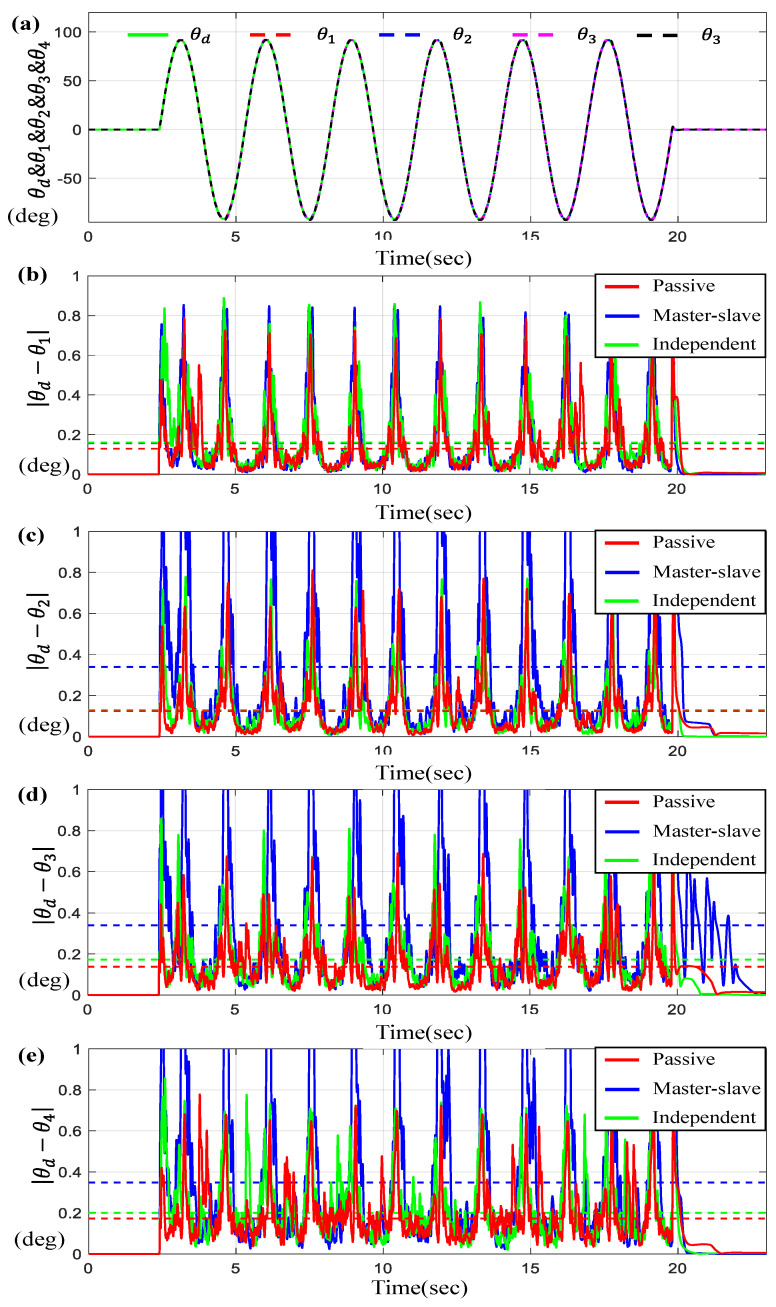
Results for four BLDC motors under sine-wave trajectory (**a**) $\theta_{d}$, $\theta_{1}$, $\;\theta_{2}$, $\theta_{3}$ and $\theta_{4}$ on the time-domain, (**b**) Error $\left|\theta_{d}-\theta_{1}\right|$, (**c**) Error $\left|\theta_{d}-\theta_{2}\right|$, (**d**) Error $\left|\theta_{d}-\theta_{3}\right|$, and (**e**) Error $\left|\theta_{d}-\theta_{4}\right|$ (the dotted lines indicate the average values).

**Figure 13:**
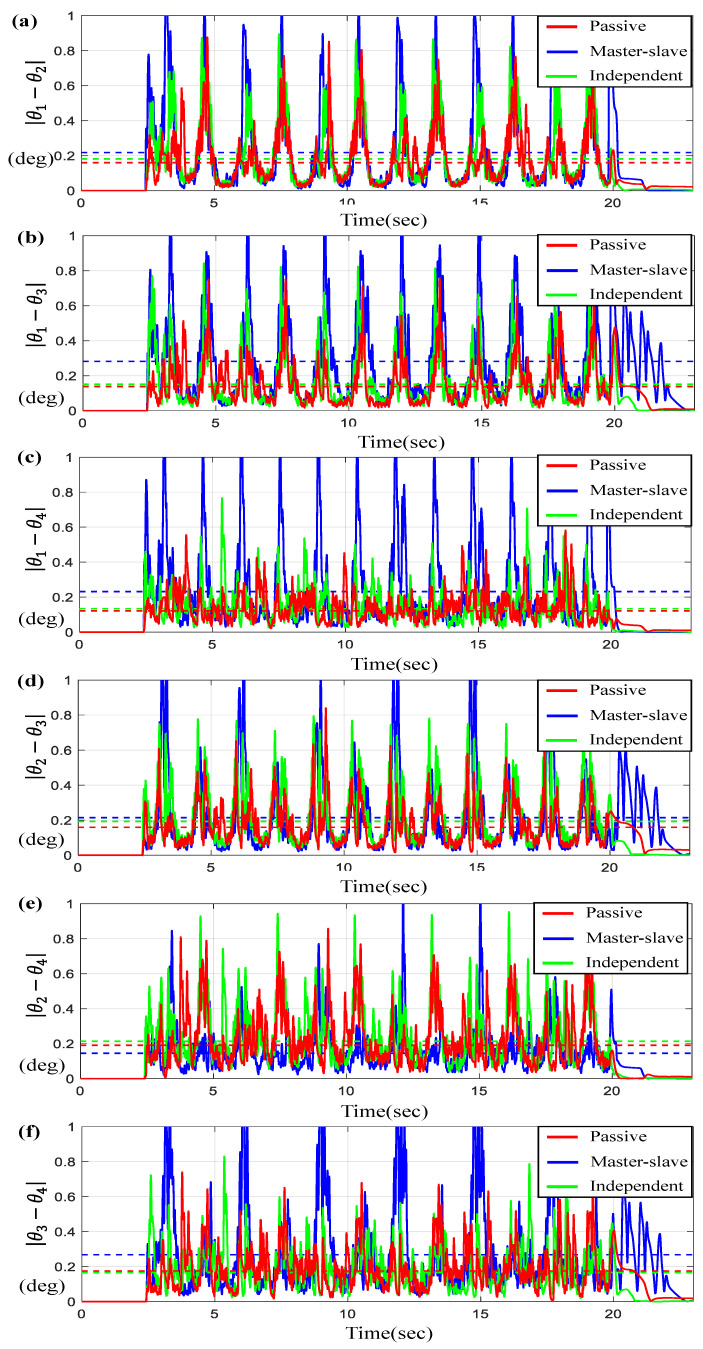
Results for four BLDC motors under sine-wave trajectory. (**a**) Error $\left|\theta_{1}-\theta_{2}\right|$, (**b**) Error $\left|\theta_{1}-\theta_{3}\right|$, (**c**) Error $\left|\theta_{1}-\theta_{4}\right|$, (**d**) Error $\left|\theta_{2}-\theta_{3}\right|$, (**e**) Error $\left|\theta_{2}-\theta_{4}\right|$, and (**f**) Error $\left|\theta_{3}-\theta_{4}\right|$ (the dotted lines indicate the average values).

For a given sine-wave trajectory scenario, Figure 12 and Figure 13 describe the tracking and synchronous performance of motors.

Figure 12a represents $\theta_{1}$, $\theta_{2}$, $\theta_{3}$, and $\theta_{4}$ along with $\theta_{d}$ on the time-domain and Figure 12b–e exhibit the tracking errors $\left|\theta_{i}-\theta_{d}\right|$ for *i* = 1, 2, 3, 4. On the other hand, Figure 13 depicts the synchronous errors $\left|\theta_{1}-\theta_{2}\right|$, $\left|\theta_{1}-\theta_{3}\right|$, $\left|\theta_{1}-\theta_{4}\right|$, $\left|\theta_{2}-\theta_{3}\right|$, and $\left|\theta_{2}-\theta_{4}\right|$ as well as $\left|\theta_{3}-\theta_{4}\right|$. Here, similar to the previous cases, you can see that the tracking and synchronous errors generated by the proposed control (“passive”) are bounded below 1 degree. Again, it can be seen from the results of Figure 12 and Figure 13 that the average tracking errors and the maximum errors via the proposed controller are the smallest among three controllers except in two cases, $\left|\theta_{2}-\theta_{4}\right|$ and $\left|\theta_{3}-\theta_{4}\right|$.

Table 5 lists the numerical values for the average tracking and the maximum errors of three controllers under the sine-wave trajectory scenario. Specifically, it is found from Table 5 that the maximum and average errors of the proposed controller (passive) are 15%~40% smaller than the errors via others.

**Figure 14:**
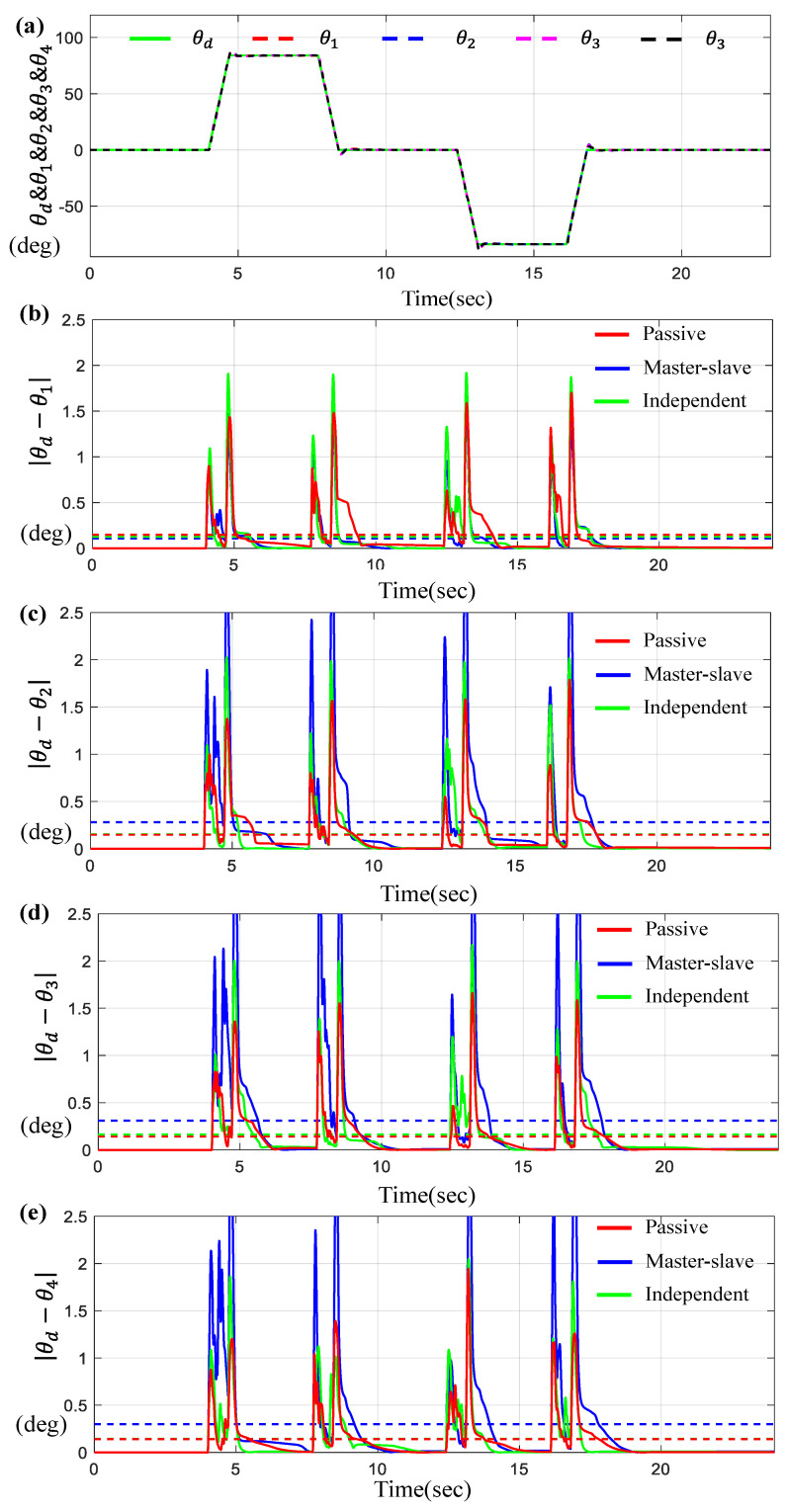
Results for four BLDC motors under trapezoidal trajectory. (**a**) $\theta_{d}$, $\theta_{1}$, $\theta_{2}$, $\theta_{3}$ and $\theta_{4}$ on the time-domain, (**b**) Error $\left|\theta_{d}-\theta_{1}\right|$, (**c**) Error $\left|\theta_{d}-\theta_{2}\right|$, (**d**) Error $\left|\theta_{d}-\theta_{3}\right|$, and (**e**) Error $\left|\theta_{d}-\theta_{4}\right|$ (the dotted lines indicate the average values).

Furthermore, Figure 14 presented the tracking and synchronous performance of motor for the trapezoidal trajectory.

**Figure 15:**
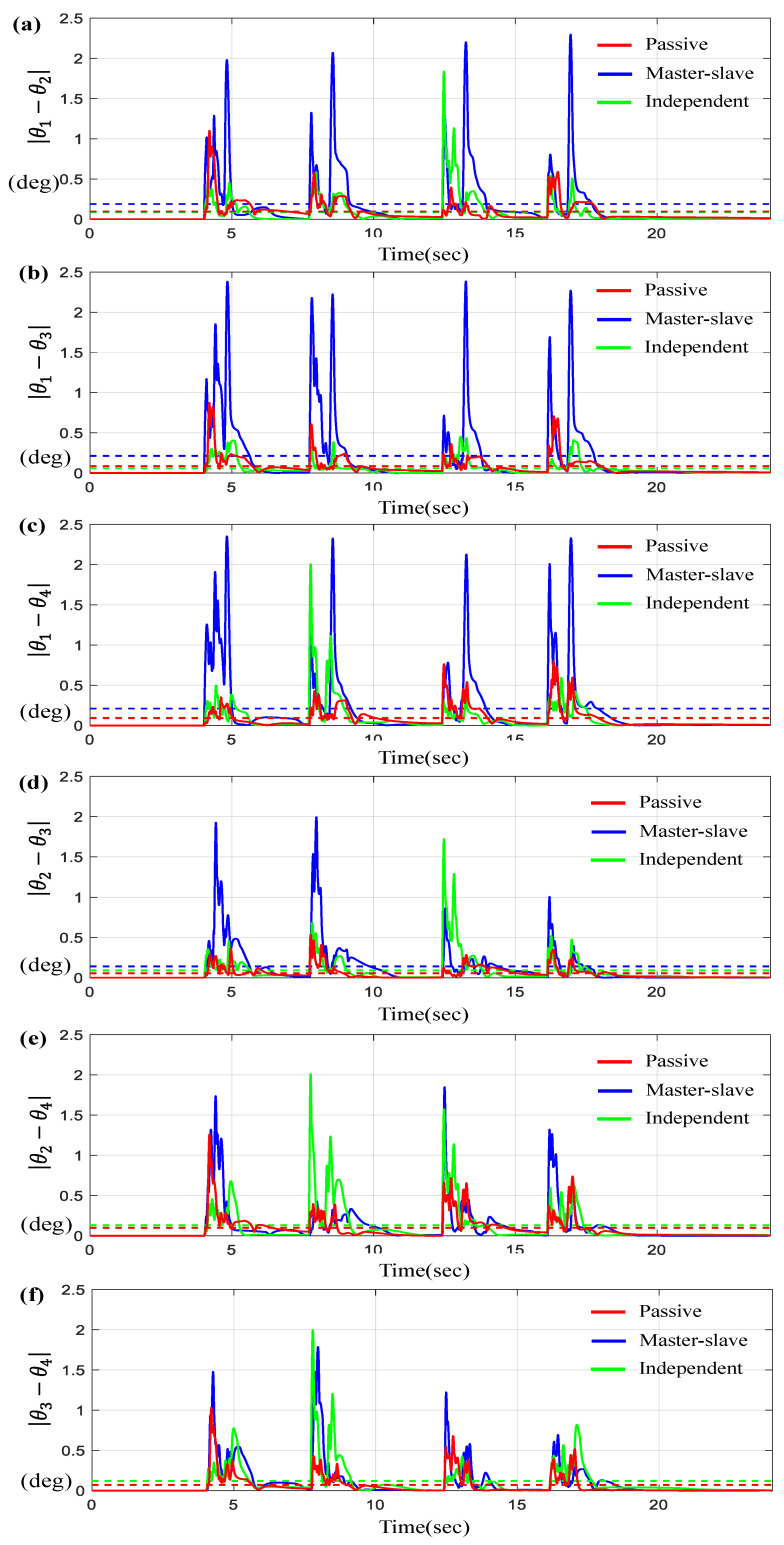
Results for four BLDC motors under trapezoidal trajectory. (**a**) Error $\left|\theta_{1}-\theta_{2}\right|$, (**b**) Error $\left|\theta_{1}-\theta_{3}\right|$, (**c**) Error $\left|\theta_{1}-\theta_{4}\right|$, (**d**) Error $\left|\theta_{2}-\theta_{3}\right|$, (**e**) Error $\left|\theta_{2}-\theta_{4}\right|$, and (**f**) Error $\left|\theta_{3}-\theta_{4}\right|$ (the dotted lines indicate the average values).

Figure 14a indicates $\theta_{1}$, $\theta_{2}$, $\theta_{3,}$ and $\theta_{4}$ for a given $\theta_{d}$ on the time-domain, and Figure 14b–e describe the tracking errors $\left|\theta_{i}-\theta_{d}\right|$ for *i* = 1, 2, 3, 4, while Figure 15 describes the corresponding synchronous errors.

As shown in Figure 14 and Figure 15, the tracking and synchronization errors through the proposed control are limited to 1.5 degrees or less, and it can be seen that the proposed control method is superior to other methods.

Table 6 shows the numerical values of the average tracking errors and the maximum errors via three controllers under trapezoidal trajectory scenario. As seen from Table 6, except for a few cases, the errors generated by the proposed control system are about 10~22% less than those of other control systems.

## 7. Conclusions

This paper presented a novel passive decomposition-based robust synchronous control system, guaranteeing that the synchronous error of the entire system is ultimately and synchronously bounded even in the presence of parametric uncertainty and external disturbance. First, a passive decomposition is utilized to achieve the locked and shape system from the original system. Second, a robust sliding mode control along with the compensation terms for disturbance and uncertainty is designed for each decomposed system to achieve the precise synchronous position control for the *n* number of agents (motors). Also, the formulation of the control law generally adopts the arbitrary *n* number of agents that must be synchronized. Finally, using two representative evaluation scenarios, a sine-wave trajectory and a trapezoidal trajectory, we validated the effectiveness of the proposed synchronous control strategy based on experimental investigation on two BLDC motors, three BLDC motors, as well as four BLDC motors, and compared the performance with that of two other well-known control approaches (a master–slave control and an independent one). It is found that the proposed system guaranteed that the synchronous error between motors and the tracking error to the desired reference trajectory are less than 1.0 degree for the sine-wave trajectory scenario and 1.5 degree for trapezoidal trajectory one, respectively. In addition, it can be seen that the synchronous tracking performance of the proposed controller is mostly superior to both the master–slave control and independent control for a sine-wave trajectory. The performance of the independent controller is a good match for the proposed controller for the trapezoidal trajectory, although the distinction between these two is almost negligible. Overall, regardless of control scenarios and the number of motors, the proposed controller guarantees a more accurate and robust synchronous control than other control methods based on the results presented in this study.

However, further investigations are still needed for how to systematically select the major control gains ($\lambda_{L}$ and $\lambda_{S}$) for each decomposed system (i.e, locked and shaped systems), because the relationship between these two control gains can influence the overall control performance of the system. We hope that the proposed control scheme along with the results and the comparisons of this study will be a valuable asset for those wishing to synchronously control a multi-motor-based system.

## Figures and Tables

**Table 1:** The average tracking and the maximum errors via three controllers under sine-wave trajectory (2 motors).

Number of Motors	Error	Value	Passive Decomposition	Master–Slave	Independent
**2**	$\left|\theta_{d}-\theta_{1}\right|$	Max	0.784	1.02	1.01
		Average	0.108	0.125	0.140
	$\left|\theta_{d}-\theta_{2}\right|$	Max	0.579	1.64	1.03
		Average	0.100	0.238	0.140
	$\left|\theta_{1}-\theta_{2}\right|$	Max	0.530	0.683	0.728
		Average	0.0715	0.164	0.114

**Table 2:** Average tracking and the maximum errors via three controllers under trapezoidal trajectory (3 motors).

Number of Motors	Error	Value	Passive Decomposition	Master–Slave	Independent
**2**	$\left|\theta_{d}-\theta_{1}\right|$	Max	1.23	1.96	1.62
		Average	0.098	0.188	0.0933
	$\left|\theta_{d}-\theta_{2}\right|$	Max	1.15	3.83	1.58
		Average	0.1	0.393	0.0996
	$\left|\theta_{1}-\theta_{2}\right|$	Max	1.35	2.17	0.478
		Average	0.131	0.260	0.0255

**Table 3:** Average tracking and the maximum errors via three controllers under sine-wave trajectory (3 motors).

Number of Motors	Error	Value	Passive Decomposition	Master–Slave	Independent
**3**	$\left|\theta_{d}-\theta_{1}\right|$	Max	0.919	1.14	0.823
		Average	0.133	0.131	0.148
	$\left|\theta_{d}-\theta_{2}\right|$	Max	0.968	2.64	1.09
		Average	0.130	0.287	0.163
	$\left|\theta_{d}-\theta_{3}\right|$	Max	0.698	2.56	1.14
		Average	0.147	0.265	0.167
	$\left|\theta_{1}-\theta_{2}\right|$	Max	0.935	1.73	1.20
		Average	0.191	0.201	0.217
	$\left|\theta_{1}-\theta_{3}\right|$	Max	0.684	1.60	0.984
		Average	0.123	0.211	0.194
	$\left|\theta_{2}-\theta_{3}\right|$	Max	0.740	0.846	0.957
		Average	0.199	0.151	0.175

**Table 4:** The average tracking and the maximum errors via three controllers under trapezoidal trajectory (3 motors).

Number of Motors	Error	Value	Passive Decomposition	Master–Slave	Independent
**3**	$\left|\theta_{d}-\theta_{1}\right|$	Max	1.32	1.76	1.53
		Average	0.098	0.156	0.0945
	$\left|\theta_{d}-\theta_{2}\right|$	Max	1.65	3.54	1.52
		Average	0.143	0.328	0.0950
	$\left|\theta_{d}-\theta_{3}\right|$	Max	1.48	3.59	1.56
		Average	0.113	0.288	0.115
	$\left|\theta_{1}-\theta_{2}\right|$	Max	0.98	1.92	0.93
		Average	0.0903	0.198	0.0371
	$\left|\theta_{1}-\theta_{3}\right|$	Max	0.596	1.93	0.874
		Average	0.0549	0.177	0.0679
	$\left|\theta_{2}-\theta_{3}\right|$	Max	1.32	1.63	0.899
		Average	0.083	0.120	0.0769

**Table 5:** The average tracking and the maximum errors via three controllers under sine-wave trajectory (4 motors).

Number of Motors	Error	Value	Passive Decomposition	Master–Slave	Independent
**4**	$\left|\theta_{d}-\theta_{1}\right|$	Max	0.956	1.071	1.07
		Average	0.129	0.158	0.160
	$\left|\theta_{d}-\theta_{2}\right|$	Max	0.985	2.59	1.10
		Average	0.124	0.340	0.129
	$\left|\theta_{d}-\theta_{3}\right|$	Max	0.818	2.53	1.02
		Average	0.137	0.340	0.172
	$\left|\theta_{d}-\theta_{4}\right|$	Max	0.983	2.64	1.24
		Average	0.173	0.348	0.200
	$\left|\theta_{1}-\theta_{2}\right|$	Max	0.876	1.83	0.895
		Average	0.159	0.218	0.181
	$\left|\theta_{1}-\theta_{3}\right|$	Max	0.758	1.80	0.843
		Average	0.138	0.282	0.151
	$\left|\theta_{1}-\theta_{4}\right|$	Max	0.582	1.88	0.766
		Average	0.122	0.232	0.134
	$\left|\theta_{2}-\theta_{3}\right|$	Max	0.839	1.45	0.863
		Average	0.160	0.214	0.193
	$\left|\theta_{2}-\theta_{4}\right|$	Max	0.857	1.11	0.953
		Average	0.191	0.145	0.214
	$\left|\theta_{3}-\theta_{4}\right|$	Max	0.740	1.50	0.830
		Average	0.175	0.268	0.166

**Table 6:** Average tracking and maximum errors via three controllers under trapezoidal trajectory (4 motors).

Number of Motors	Error	Value	Passive Decomposition	Master–Slave	Independent
**4**	$\left|\theta_{d}-\theta_{1}\right|$	Max	1.64	1.57	1.70
		Average	0.128	0.107	0.113
	$\left|\theta_{d}-\theta_{2}\right|$	Max	1.51	3.48	1.79
		Average	0.138	0.281	0.137
	$\left|\theta_{d}-\theta_{3}\right|$	Max	1.52	3.61	1.92
		Average	0.142	0.310	0.144
	$\left|\theta_{d}-\theta_{4}\right|$	Max	1.96	3.68	2.11
		Average	0.13	0.298	0.128
	$\left|\theta_{1}-\theta_{2}\right|$	Max	1.21	2.29	1.62
		Average	0.1	1.88	0.0979
	$\left|\theta_{1}-\theta_{3}\right|$	Max	0.81	2.38	0.401
		Average	0.0873	0.212	0.0820
	$\left|\theta_{1}-\theta_{4}\right|$	Max	0.711	2.35	1.77
		Average	0.081	0.211	0.0834
	$\left|\theta_{2}-\theta_{3}\right|$	Max	0.411	1.99	1.62
		Average	0.0634	0.141	0.0797
	$\left|\theta_{2}-\theta_{4}\right|$	Max	1.44	1.85	1.98
		Average	0.104	0.131	0.115
	$\left|\theta_{3}-\theta_{4}\right|$	Max	1.08	1.79	1.97
		Average	0.0793	0.122	0.126

## Appendix A

This section briefly explains the advantageous effect of integral error terms in a sliding surface when it comes to designing the sliding mode control.

The dynamics of a motor are given by

(A1) $$J_{R}{\ddot{\theta }}_{R}\left(t\right)+B_{R}{\dot{\theta }}_{R}\left(t\right)=\tau_{m,R}\left(t\right)+d_{R}$$

Proposing the following control law including the integral error term,

(A2) $$\tau_{m,R}=B_{R}{\dot{\theta }}_{R}+J_{R}({\ddot{\theta }}_{R,d}-\left(3\lambda \dot{\varepsilon }+3\lambda^{2}\varepsilon +\lambda^{3}\int \varepsilon dt\right))$$

where $\varepsilon =\theta_{R}-\theta_{R,d}\in R$ is the error between the desired trajectory ($\theta_{R,d}$) and an actual angle ($\theta_{R}$). Also, $S_{R}\left(t\right)=\dot{\varepsilon }+2\lambda \varepsilon +\lambda^{2}\int \varepsilon \;dt\in R$ is the designed sliding surface.

Furthermore, substituting (A2) into (A1) yields the closed-loop system as follows,

(A3) $$\ddot{\varepsilon }=-\left(3\lambda \dot{\varepsilon }+3\lambda^{2}\varepsilon +\lambda^{3}\int \varepsilon dt\right)+d_{R}$$

Based on Equation (A3), the transfer function $T(s)=\varepsilon (s)/d_{R}(s)$ becomes

(A4) $$T(s)=\varepsilon (s)/d_{R}(s)=1/(s^{3}+3\lambda s^{2}+3\lambda^{2}s+\lambda^{3})$$

Using the final-value theorem, we can find the DC gain of ε(s) for $d_{R}\left(s\right)=1/s$ (i.e., coulomb-like friction disturbance) such that

(A5) $$\varepsilon_{ss}=\underset{s=0}{\lim}s\left[\frac{1}{s^{3}+3\lambda s^{2}+3\lambda^{2}s+\lambda^{3}}\right]\frac{1}{s}=1/\lambda^{3}$$

The steady-state error $\varepsilon_{ss}$ for the disturbance $d_{R}\left(s\right)$ is attenuated by $\lambda^{3}$ as shown in (A5).

On the other hand, if the sliding surface is designed to be $S_{R}\left(t\right)=\dot{\varepsilon }+\lambda \varepsilon \in R$ (i.e., w/o an integral term), a control law similar to the previous case can be proposed by

(A6) $$\tau_{m,R}=B_{R}{\dot{\theta }}_{R}+J_{R}({\ddot{\theta }}_{R,d}-\left(2\lambda \dot{\varepsilon }+\lambda^{2}\varepsilon \right))$$

The closed-loop system is given by

(A7) $$\ddot{\varepsilon }=-\left(2\lambda \dot{\varepsilon }+\lambda^{2}\varepsilon \right)+d_{R}$$

Based on (A7), the transfer function $T(s)=\varepsilon (s)/d_{R}(s)$ becomes

(A8) $$T(s)=\varepsilon (s)/d_{R}(s)=1/(s^{2}+2\lambda s+\lambda^{2})$$

Using the final-value theorem, we can find the DC gain of ε(s) to $d_{R}\left(s\right)$ as

(A9) $$\varepsilon_{ss}=\underset{s=0}{\lim}s\left[1/(s^{2}+2\lambda s+\lambda^{2})\right]\frac{1}{s}=1/\lambda^{2}$$

The steady-state error $\varepsilon_{ss}$ for the disturbance $d_{R}\left(s\right)$ is attenuated by $\lambda^{2}$ as shown in (A9).

The controller designed based on the sliding surface including the integral error term attenuates the disturbance by $\lambda^{3}$ while the case w/o integral term reduces the disturbance effect by $\lambda^{2}$.
